# Insights into Hypoxic Systemic Responses Based on Analyses of Transcriptional Regulation in *Arabidopsis*

**DOI:** 10.1371/journal.pone.0028888

**Published:** 2011-12-15

**Authors:** Fu-Chiun Hsu, Mei-Yi Chou, Hsiao-Ping Peng, Shu-Jen Chou, Ming-Che Shih

**Affiliations:** 1 Agricultural Biotechnology Research Center, Academia Sinica, Taipei, Taiwan; 2 Department of Biology, University of Iowa, Iowa City, Iowa, United States of America; 3 Institute of Plant and Microbial Biology, Academia Sinica, Taipei, Taiwan; Michigan State University, United States of America

## Abstract

We have adopted a hypoxic treatment system in which only roots were under hypoxic conditions. Through analyzing global transcriptional changes in both shoots and roots, we found that systemic signals may be transduced from roots to trigger responses in tissues not directly subjected to hypoxia. The molecular mechanisms of such systemic responses under flooding are currently largely unknown. Using ontological categorization for regulated genes, a systemic managing program of carbohydrate metabolism was observed, providing an example of how systemic responses might facilitate the survival of plants under flooding. Moreover, a proportion of gene expressions that regulated in shoots by flooding was affected in an ethylene signaling mutation, *ein2-5*. Many systemic-responsive genes involved in the systemic carbohydrate managing program, hormone responses and metabolism, ubiquitin-dependent protein degradation were also affected in *ein2-5*. These results suggested an important role of ethylene in mediation of hypoxic systemic responses. Genes associated with abscisic acid (ABA) biosynthesis are upregulated in shoots and down regulated in roots. An ABA signaling mutation, *abi4-1*, affects expression of several systemic responsive genes. These results suggested that regulation of ABA biosynthesis could be required for systemic responses. The implications of these results for the systemic responses of root-flooded *Arabidopsis* are discussed.

## Introduction

With global climate changes, flooding has become a more important issue around the world. Flooding reduces plant survival or impedes plant growth, and causes serious yield losses in crops. Rice is a semi-aquatic plant that can tolerate hypoxic stress in roots. Submergence-tolerant rice cultivars develop a quiescence strategy that restrains consumption of carbohydrates and elongation of stems and leaves through regulating expression of an ethylene-responsive factor (ERF) gene, *Sub1A* [1], [2]. Deepwater rice cultivars have evolved an escape strategy that involves fast elongation of stems and leaves according to the depth of water under the regulation of two ERFs, *Snorkel1* (*SK1*) and *Snorkel2* (*SK2*) [3]. Unlike rice, waterlogging of the root system is sufficient to cause damage of the whole plant in most land plant species. For these species, waterlogging of roots should resemble the condition in nature under which hypoxia adaptation evolved, whereas submergence of whole plants could be too harsh to exhibit the adaptations they evolved. However, few of these adaptations have been studied in these non-wetland plants such as maize, tomato, and *Arabidopsis*. Consequently, it remains unclear what the molecular basis of hypoxic responses is in both aerial parts and roots under waterlogging of roots.

When roots were subjected to flooding, some signals could convey from roots to shoots. It was shown that 1-aminocyclopropane-1-carboxylic acid (ACC), the precursor of ethylene, serves as a signal from roots to shoots which results in petiole epinasty in waterlogged tomato [4], [5]. Accumulation of leaf abscisic acid (ABA) could be partially responsible for stomatal closure in root-flooded tomato, but other unknown factors in xylem sap actively close stomata [6]. Not only has an increase in the level of ABA in leaves been reported when roots is flooded, but accumulation of both ABA and jasmonic acid (JA) in leaves is also observed in flooded citrus [7]. These results suggest that these hormones may trigger downstream responses in shoots when roots are subjected to hypoxia. However, little is known about how these systemic responses are initiated and what the molecular bases are in these systemic responses.

Various sets of hypoxia-responsive transcription factors (TFs) have recently been shown to regulate expression of hypoxic core genes [8]. A myeloblastosis (MYB) TF, MYB2, is known to be involved in the induction of *ALCOHOL DEHYGROGENASE 1* (*ADH1*) under conditions of low oxygen [9]. A NAC (NAM/ATAF/CUC) TF, ANAC102, affects seed viability under hypoxic conditions [10]. AP2 (APETALA2)/ERFs are also recently shown to affect hypoxia responses [11]–[13]. However, so far no TF has been assigned a role in systemic responses in non-flooded tissues under waterlogging.

Many published studies of hypoxia were carried out with a hypoxic solution or a low-oxygen atmosphere [1], [3], [14]–[16], but have overlooked systemic response because the entire plants or the cultured tissues were subjected to hypoxic conditions. Only two recent studies provided transcriptomic analyses in waterlogged gray poplar and cotton [17], [18]. No significant transcriptional regulation is observed in shoots of waterlogged gray poplar [18]. Genes that are associated with cell wall synthesis and tetrapyrrole synthesis were regulated in leaves of cotton seedlings after 24 hours of flooding [17]. However, none of these studies has discussed regulation of systemic responses under flooding.

To gain insights into systemic responses under flooding, we investigated global transcriptional changes in both shoots and roots in *Arabidopsis* with only roots under hypoxic conditions. We found that distinct classes of genes are regulated in shoots and roots. Using ontological categorization, we deduced a model that carbohydrates are managed by a systemic response to generate carbohydrates in shoots and transport to roots for supporting the extreme energy crisis in flooded roots. We demonstrate that many genes that are specifically regulated in roots are tissue autonomous, while genes that are specifically regulated in shoots, including several classes of TFs, are mediated by systemic signals from roots. We also found that DNA-binding motifs corresponding to the TFs are present in the promoters of systemic responsive genes. Furthermore, expression of local and systemic responsive genes was globally affected in an ethylene-insensitive mutant (*ein2-5*) and showed ethylene plays an important role in systemic responses. Transcriptional regulation of ABA biosynthesis could also play a role in systemic responses. On the basis of our results, we discuss the role of systemic response in partial flooded *Arabidopsis* plants and what factors are associated in the regulation of the systemic responses. Although the details of underlying signaling and regulatory components are not determined in the work, we establish a completely new branch of hypoxic responses and provide a series of time course and *ein2-5* mutant transcriptomic datasets to the scientific community.

## Results

### Flooding of roots not only triggers hypoxic responses in roots but also systemic responses in shoots

**Figure 1:**
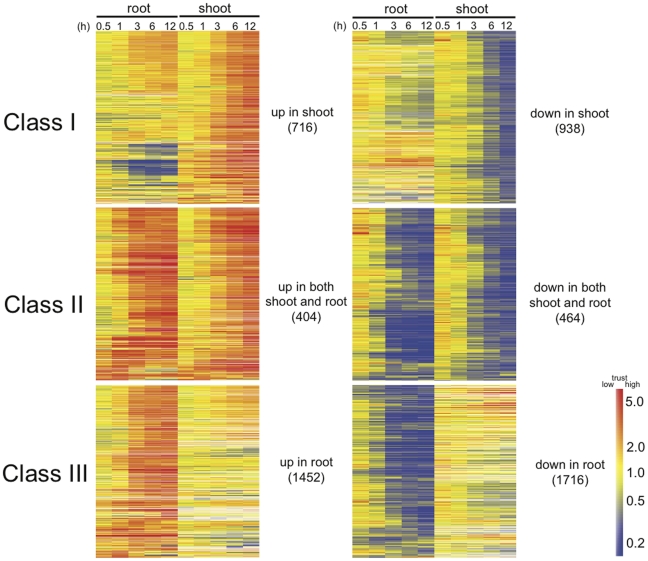
Shoot and root-specific genes responsive to root flooding. Fourteen-day-old Columbia plants were root flooded for up to 12 hours. Shoots and roots were collected at specific time points (0, 0.5, 1, 3, 6, and 12 h). Total RNA was analyzed by microarray analysis with the 0 hour time point as the control channel. Three classes of genes were filtered by >2-fold change (upregulated) or <0.5-fold change (downregulated) expression with *p* values<0.05 at any one time point from 1 to 12 h in shoot and/or root. Class I, regulated in shoot only; Class II, regulated in both shoot and root; Class III, regulated in root only. Gene Tree Clustering was performed using the GeneSpring software package by Pearson Correlation similarity measure and Average Linkage clustering algorithm from each class of gene list. The numbers in parentheses indicate the number of genes in each class. Color scale indicates treatment-to-control ratios of expression. A lighter color in the scale indicates lower trust with a higher *p* value. Corresponding datasets can be found in Supporting Information File S1.

To better understand how an entire plant responds to hypoxia in roots, we established an open system in which only root is subjected to hypoxia treatment. Through microarray studies, we observed transcriptomic kinetic profiling in shoots and roots. We identified three different classes of genes that are regulated during flooding: genes that are regulated only in shoots (Class I); genes that are regulated in both roots and shoots (Class II); and genes that are regulated only in roots (Class III) (Figure 1, File S1). These three classes were filtered out at fold change >2 and <0.5 in expression with *p* values<0.05 at any one time point from 1 to 12 h in shoots and/or roots. The existence of Class I and II genes suggests that hypoxia triggers signals that travel from roots to shoots to regulate systemic responses, while the existence of Class II and III genes in roots demonstrates local responses to low oxygen levels.

### Regulated genes fall into distinct functional classes in shoots and roots

To compare the transcriptomic responses between shoots and roots, we first investigated which metabolic processes were altered during hypoxia. To accomplish this, global gene expression profiles in shoots and roots after 6 hours of root flooding treatment were analyzed via Wilcoxon Rank Sum test with a Benjamini-Yekutieli correction in Mapman software [19], [20] (Fig. S1). As anticipated, genes associated with the fermentation pathway were increased in roots, but not in shoots, indicating that fermentation is only induced in the tissues directly subjected to flooding. Moreover, genes associated with other metabolic pathways were also affected distinctly between shoots and roots. For example, genes associated with cell wall synthesis and modification (*p*<0.01), flavonoid production (*p*<0.05), and cell wall degradation (*p*<0.05) were mostly downregulated in roots, while these genes were not significantly regulated in shoots. By contrast, genes associated with photosynthetic light reactions (*P*<0.01), tetrapyrrole synthesis (*p*<0.01), and the Calvin cycle (*p*<0.05) were significantly downregulated in shoots. Thus, these results show that global gene expression in shoots is distinctly regulated under root hypoxia.

To further dissect the spectrum of affected metabolic pathways, we used an *Arabidopsis* web-based classification tool, Functional Classification SuperViewer [21], to investigate pathways that are significantly regulated in different ontological categories. Over-represented functional categories in gene lists of Class I, II, and III were identified, respectively. Comparing these categories among the three classes revealed similarities and differences of regulation between shoots and roots. Table 1 shows the normalized frequencies of functional categories in each class. Bold text indicates a significantly over-represented category (with a *p* value<0.05). Genes in the functional categories of major carbohydrate (CHO) metabolism, nucleotide metabolism, gluconeogenesis/glyoxylate cycle, lipid metabolism, and secondary metabolism were significantly over-represented in Classes I and II but not in Class III (Table 1) in the list of upregulated genes, suggesting that genes associated with these functional categories are mainly upregulated in shoots. On the contrary, genes in functional categories of minor CHO metabolism, cell signaling, stress, photosynthesis, and fermentation were primarily upregulated in roots under flooding (Table 1). Moreover, in the list of downregulated genes, those in the functional categories of tetrapyrrole synthesis, pentose phosphate pathway, N-metabolism, S-assimilation, minor CHO metabolism, and photosynthesis were significantly over-represented in Class I and Class II, whereas those involved in cell wall synthesis, stress, metal handling, signaling, development, glycolysis, and biodegradation of xenobiotics were over-represented in Class II (Table 2).

**Table 1:** Over-represented functional categories in upregulated gene lists.

		Class I shoot only (717 genes)		Class II both shoot and root (404 genes)		Class III root only (1452 genes)	
Functional category	Number of genes in category^a^	Freq.^b^	Number of gene	Freq.^b^	Number of gene	Freq.^b^	Number of gene
major CHO metabolism	102	**2.75**	6	2.44	3	1.13	5
nucleotide metabolism	180	**2.6**	10	1.38	3	1.15	9
gluconeogenesis/glyoxylate cycle	13	**10.81**	3	**19.14**	3	1.78	1
lipid metabolism	415	**1.92**	17	**2.19**	11	1.17	21
secondary metabolism	442	**1.59**	15	**2.06**	11	0.89	17
miscellaneous	1595	**2.05**	70	**1.97**	38	**1.32**	91
amino acid metabolism	258	**1.81**	10	**5.14**	16	**1.97**	22
not assigned	11832	**0.93**	236	**0.93**	133	**0.99**	510
protein	4674	**0.92**	92	**0.71**	40	**0.81**	164
RNA	3101	**0.86**	57	**1.25**	47	**1.08**	145
DNA	3135	**0.08**	6	**0.15**	6	**0.12**	17
metal handling	89	**3.16**	6	1.86	2	**2.08**	8
transport	1044	**2.15**	48	1.11	14	**1.15**	52
development	791	**2.01**	34	0.62	6	**1.84**	63
hormone metabolism	534	**1.66**	19	0.93	6	**1.82**	42
minor CHO metabolism	124	1.51	4	**4.68**	7	**2.24**	12
biodegradation of xenobiotics	28	3.34	2	**5.92**	2	0	0
cell	824	1.02	18	0.9	3	**0.64**	23
signalling	1365	0.82	24	0.72	12	**1.47**	87
stress	1223	0.95	25	1.28	19	**1.57**	83
photosynthesis	204	0	0	0	0	**2.15**	19
fermentation	14	3.34	1	0	0	**9.92**	6

Bold text indicates a significantly over-represented category (with a *P* value<0.05).

a Number of genes in the category in whole genome.

b Normalized Frequency is calculated as follows: (Number_in_Class_input_set_/Number_Classified_input_set_)/(Number_in_Class_reference_set_/Number_Classified_reference_set_).

Upregulated genes from each class were classified with an *Arabidopsis* web-based classification tool, Functional Classification SuperViewer [21] via Mapman category. Functional categories with *P* values<0.05 in at least one class are shown in the table.

**Table 2:** Over-represented functional categories in downregulated gene lists.

		Class I shoot only (938 genes)		Class II both shoot and root (464 genes)		Class III root only (1716 genes)	
Functional category	Number of genes in category^a^	Freq.^b^	Number of gene	Freq.^b^	Number of gene	Freq.^b^	Number of gene
tetrapyrrole synthesis	48	**11.2**	14	0	0	0	0
pentose phosphate pathway	32	**6.7**	6	0	0	1.8	3
N-metabolism	26	**4.1**	3	**5.6**	2	0	0
S-assimilation	13	**5.5**	2	**22.3**	4	0	0
minor CHO metabolism	124	**2.0**	7	**3.5**	6	1.6	10
photosynthesis	204	**8.8**	50	**2.8**	8	**0.4**	4
amino acid metabolism	258	**3.2**	23	**2.8**	10	**1.7**	22
nucleotide metabolism	180	**3.0**	15	**3.2**	8	**1.7**	16
redox	210	**2.4**	14	**2.1**	6	**1.5**	16
hormone metabolism	534	**2.6**	39	**2.6**	19	**2.5**	69
protein	4674	**1.0**	131	**0.7**	45	**0.5**	122
RNA	3101	**0.9**	81	**0.6**	26	**0.9**	139
not assigned	11832	**0.8**	265	**0.6**	97	**0.7**	433
DNA	3135	**0.3**	23	**0.0**	2	**0.1**	12
transport	1044	**0.7**	21	**1.5**	21	**2.4**	126
lipid metabolism	415	**1.4**	16	**1.9**	11	**2.3**	49
miscellaneous	1595	**1.3**	58	**2.3**	51	**2.6**	211
secondary metabolism	442	**1.9**	24	**4.3**	26	**3.8**	86
cell	824	**1.4**	33	1.0	11	**0.8**	35
C1-metabolism	40	0	0	**7.2**	4	0.5	1
major CHO metabolism	102	1.8	5	**3.5**	5	1.3	7
cell wall	536	1.1	16	**5.5**	41	**3.1**	84
stress	1223	0.9	30	**2.1**	36	**1.4**	86
metal handling	89	0.8	2	**4.1**	5	**2.4**	11
signalling	1365	1.1	42	0.9	17	**1.2**	82
development	791	0.8	17	1.0	11	**1.7**	70
glycolysis	75	1.9	4	1.9	2	**3.9**	15
biodegradation of xenobiotics	28	0	0	0	0	**3.5**	5

Bold text indicates a significantly over-represented category (with a *P* value<0.05).

a Number of genes in the category in whole genome.

b Normalized Frequency is calculated as follows: (Number_in_Class_input_set_/Number_Classified_input_set_)/(Number_in_Class_reference_set_/Number_Classified_reference_set_).

Downregulated genes from each class were classified with an *Arabidopsis* web-based classification tool, Functional Classification SuperViewer [21]. Functional categories with *P* values<0.05 in at least one class are shown in the table.

### Flooding of roots induces comprehensive systemic regulation of carbohydrate metabolism

**Figure 2:**
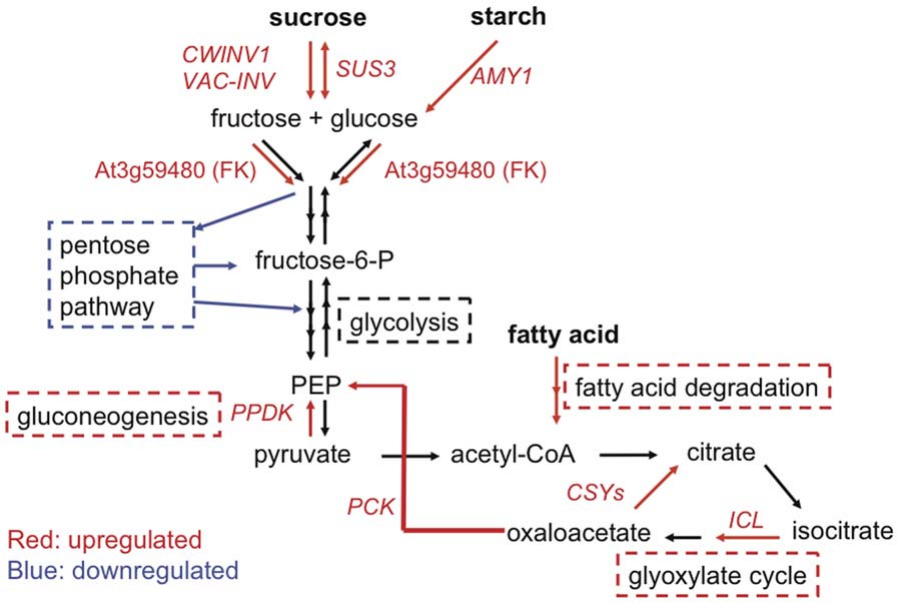
Schematic model of the hypoxic systemic regulation of carbohydrate metabolism. *Arabidopsis* transcriptionally regulates systemic responses of multiple metabolic pathways to generate carbohydrates from starch, sucrose, and fatty acid in tissues not directly subjected to hypoxia during partial flooding. Red arrows and dashed boxes indicate reactions and metabolic pathways that are promoted by upregulating associated genes, whereas blue signifies downregulations. Gene abbreviations are as follows: PEP, *PHOSPHOENOLPYRUVATE*; *CWINV1*, *CELL WALL INVERTASE 1*; *VAC-INV1*, *VACUOLAR INVERTASE*; *SUS3*, *SUCROSE SYNTHASE* 3; *AMY1*, *ALPHA-AMYLASE*; At3g59480 (*FK*), encoding pfkB-type carbohydrate kinase family protein (fructokinase); *PPDK*, *PYRUVATE ORTHOPHOSPHATE DIKINASE*; *PCK*, *PHOSPHENOLPYRUVATE CARBOXYLASE KINASE*; *CSYs*, *CITRATE SYNTHASES*; *ICL*, *ISOCITRATE LYASE*.

To gain insights into why many functional categories were regulated in shoots when only roots were under flooding, we looked into functions of pathways and genes within these functional categories. For example, we profiled the regulation of genes that are associated with carbohydrate metabolism and related functional categories (Tables S1, 2) by Q-RT-PCR (Table S3), as this may provide clues to possible explanations for an alternative energy usage program under root flooding. Notably, in the category of major CHO metabolism in the Class I list, except *α-AMYLASE 1*, all other five genes that encode enzymes for sucrose degradation, i.e., three invertases, a sucrose synthase, and a pfkB-type carbohydrate kinase were up regulated (Table 1, Tables S1, S3), indicating induction of starch breakdown and sucrose degradation. Moreover, the six genes in the Class I list that are associated with the pentose phosphate pathway were downregulated (Table 2, Tables S2, S3). These results suggest that flooding triggers sucrose degradation and that the produced monosaccharides might primarily go into glycolysis instead of shunt into the pentose phosphate pathway in shoots of root-flooded *Arabidopsis* (Figure 2). Additionally, six out of thirteen genes associated with gluconeogenesis/glyoxylate cycle were upregulated in shoots (three in Class I and three in Class II; Table 1). It has been shown that the glyoxylate cycle is primarily involved in the conversion of lipids to carbohydrates [22]–[25]. The category of lipid metabolism was over-represented in Class I (Table 1), and eight out of seventeen genes in this category were assigned in lipid degradation (Table S1). This result raised the possibility that lipids could be converted into carbohydrates in shoots of root-flooded *Arabidopsi*s (Figure 2). Kreuzwieser *et al.* (2009) have shown that sucrose does not accumulate in leaves, but accumulates in roots of root-flooded poplar, implicating that sucrose, the major form of carbohydrates, is transported from leaves to roots in flooded plants. In our study, we observed a higher frequency of upregulated genes that function as carbohydrate transporters in shoots (9 out of 716 genes) than in roots (4 out of 1452 genes), while the frequency of these genes that were downregulated was higher in roots (12 out of 1716 genes) than in shoots (1 out of 938 genes) under flooding (Table S4). Taken together, these results suggest that carbohydrates could be transported from shoots to roots.

Regarding the Q-RT-PCR validation, genes that were manually selected from microarray results (Table S1 and S2) were tested by Q-RT-PCR, and are shown in Table S3. Trends of seventeen up or downregulated genes in Q-RT-PCR were consistent with those in microarray data except the induction of one gene, At4g14430; hence, the regulation of ∼94% tested genes was validated by Q-RT-PCR. To distinguish the flooding-specific effects, non-flooded controls was collected parallel with flooded samples. Similar or weaker regulation of some genes was also observed in non-flooded controls. These genes included two genes in the upregulated list, At3g59480 and At3g58750, and five genes in the downregulated list, At5g18660, At3g51820, At4g09650, At1g52230, and At3g54050. This non-specific regulation of these genes was primarily in the categories of tetraphyrrole synthesis and photosynthesis (Table S3); therefore, I consider these two categories as uncorrelated with systemic responses of flooding.

### Root-responsive genes under flooding are primarily hypoxic core genes and tissue autonomous responsive

**Figure 3:**
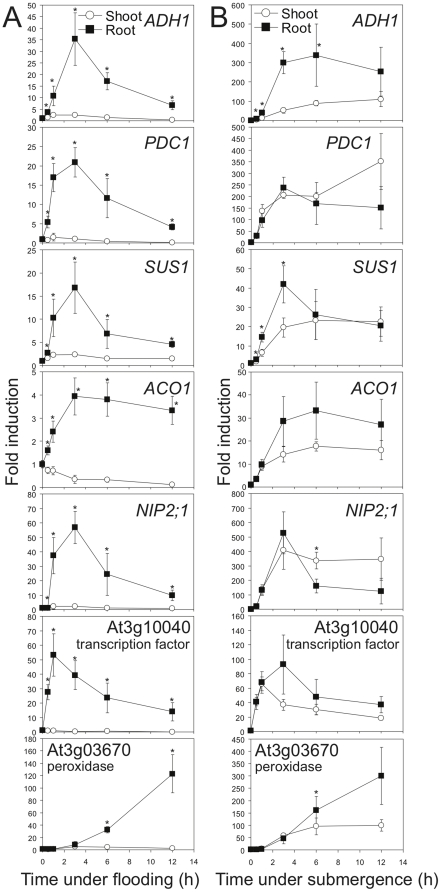
Root-specific genes regulated by flooding are largely tissue autonomous. (A) Induction of hypoxic core genes in roots under flooding of roots. (B) Induction of hypoxic core genes in both shoots and roots under submergence. Fourteen-day-old Columbia plants were root flooded or entirely submerged for up to 12 hours. Shoot and root tissues were collected at specific time points (0, 0.5, 1, 3, 6, and 12 h). Total RNA was analyzed by Q-RT-PCR using gene-specific primers. The level of *Tubulin* mRNA was used as an internal control. The data represent means ± S.D. from six independent biological replicates, and an asterisk indicates a significant difference between shoots and roots (P<0.05). Gene details are shown in the Supporting Information Table S10.

The root-specific response in Class III genes (i.e., genes specifically regulated in roots) could be mainly caused by two mechanisms: (1) tissue specificity, or (2) tissue autonomous response in the tissues that were under hypoxia. To determine which mechanism contributes to the root-specific response, we established a submergence system that provides hypoxic conditions for both shoots and roots. If Class III genes are responsive in both shoots and roots under submergence, it is likely that they are tissue autonomous responsive under hypoxia. We first determined the expression of well-known hypoxic core genes, including *ALCOHOL DEHYDROGENASE 1* (*ADH1*), *PYRUVATE DECARBOXYLASE 1* (*PDC1*), *SUCROSE SYNTHASE 1* (*SUS1*) and *ACC OXYDASE 1* (*ACO1*). All these hypoxic core genes were dramatically induced in roots but not in shoots of root-flooded plants (Figure 3a). By contrast, these genes were induced in both shoots and roots under submergence (Figure 3b), indicating that the induction of these genes is a tissue-autonomous response.

To further confirm the tissue-autonomous response under hypoxia, we determined the expression of novel hypoxia-responsive genes that are strongly induced only in roots under flooding by Q-RT-PCR. Two of the novel marker genes, which are a gene encoding a putative water channel, NOD26-like intrinsic protein 2;1 (NIP2;1), and a gene encoding a putative transcription factor (At3G10040), were reported to be induced during hypoxia based on microarray analyses [15], [26], [27]. These marker genes as well as another novel marker gene encoding a putative peroxidase (At3g03670) showed strong induction in roots under flooding (Figure 3a). Regardless of the distinct induction patterns of these novel markers, all of them were induced when shoots were also subjected to hypoxia (Figure 3), indicating that these inductions are tissue-autonomous responses to hypoxia. Thus, the induction of known hypoxic core genes and novel hypoxia markers that are induced only in roots under flooding is a tissue-autonomous response. Moreover, many genes regulated by hypoxic core responses are present in the Class III list (Figure 1), including many *HYPOXIA-RESPONSIVE UNKNOWN PROTEIN* (*HUP*) genes [14]. Taken together, these results suggest that Class III genes are primarily under the control of tissue-autonomous regulations, which could also reflect so-called hypoxic core responses.

The induction level of each core hypoxia-responsive gene is 3–10 times greater in roots under completely submergence than partial submergence (Figure 3). It is likely that complete submergence could contribute higher tension of energy crisis in roots than partial submergence to cause the higher induction. Integrated transcription networks of stress and energy signaling share part of their signaling pathways, suggesting that sensing of energy deprivation could increase stress responses, and *vice versa* [28]; thus, this strengthen the idea that hypoxia plus a stronger energy crisis could induce higher levels of hypoxia-responsive genes under complete submergence. This explanation also agrees with our model for systemic regulation of carbohydrates. In the model, we suggested that shoots could be a source of energy for roots under partial submergence. Under complete submergence, shoots are also under hypoxia, so roots could be under a more serious energy crisis to cause the higher induction than under partial submergence.

### Transcription factors are regulated to mediate systemic responses

**Figure 4:**
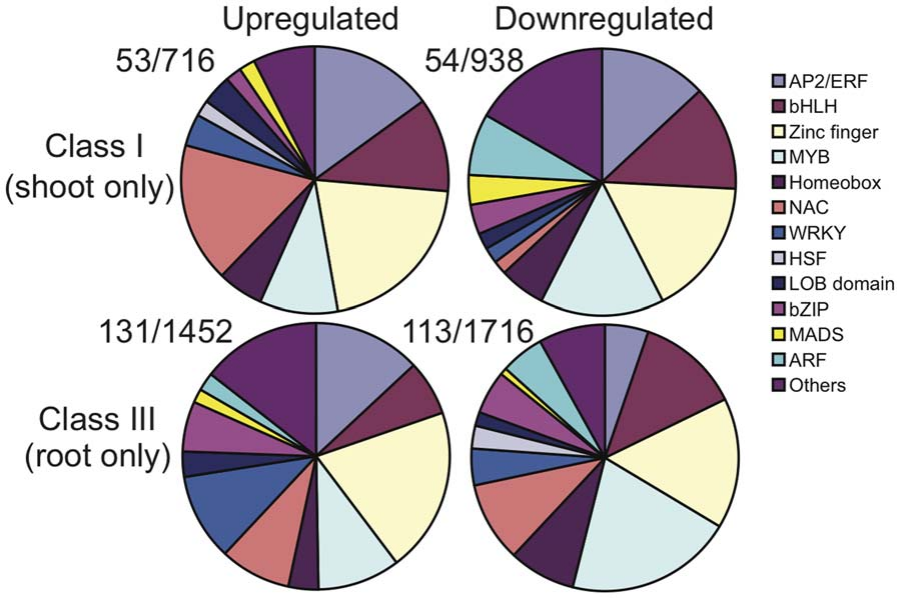
Subsets of transcription factors (TFs) were differentially expressed in shoots and roots under root flooding. Pie chart depicting the proportion of regulated TF genes in protein families. Class I, genes regulated in shoots only; Class III, genes regulated in roots only. Upregulated and downregulated indicates up- or downregulated genes in either Class I or Class III. Ratio indicates TF number/total gene number in each class.

Various sets of TFs have been identified to mediate expression of the hypoxic core genes [8]. To identify TFs that are involved in systemic responses, we first identified differentially expressed TFs in shoots of root-flooded plants. Fifty-three TFs were upregulated and fifty-four TFs were downregulated in Class I, indicating the involvement of TFs in the systemic response under flooding. We then classified these TFs according to their assigned protein families (Figure 4). By comparing up- and downregulated sets of TFs within Class I, we found that AP2/ERF, bHLH (Basic Helix-Loop-Helix), and Zinc-finger TFs were common types in both sets. MYB and ARF (Auxin Response Factor) TFs were mainly present in the downregulated set, whereas NAC TFs were primarily present in the upregulated set. To identify the types of TFs mainly involved in systemic response, we compared the proportion of these sets of TFs between Class I and Class III (Figure 4). AP2/ERF TFs were present in systemic response (Class I), with an about equal fraction up- or downregulated, whereas in hypoxic core response (Class III), they were primarily upregulated. As described previously, NAC TFs were primarily upregulated in shoot. However, in hypoxic core response, NAC TFs were common types of TFs in both up- and downregulation. Moreover, WRKY TFs were a type that appeared in both Class I and Class III; however, they were only enriched in the upregulated set in hypoxic core response (Class III). Next, we used Q-RT-PCR to confirm the regulation of selected TFs. Two AP2/ERFs, *NAC089*, *MYB2*, *HSFC1* (Heat Shock Factor), and *ATHB-7* (Homeobox-7) were significantly upregulated in shoots under flooding in agreement with microarray data (Table S5).

To assess the binding potential of long-distance responsive TFs to promoters of long-distance responsive genes, we analyzed the enrichment of corresponding *cis*-elements. Stringent lists of systemic responsive up- or down-regulated genes were filtered out from up- or downregulated genes in Class I at fold change >2 or <0.5 in expression with *p* value<0.02 or 0.025 and raw intensity >300 at 3 hour-flooding time point, respectively. Class I lists was filtered down to seventy genes in the upregulated list and to sixty-three genes in the downregulated list. The upstream 500 nucleotides of the filtered genes were extracted for scanning over-represented elements by the Motif Analysis on The Arabidopsis Information Resource (TAIR) website (Tables S6, S7). Five families of TFs were identified that are potentially able to bind *cis*-elements on the promoter of at least half of the genes in the stringent gene lists. The ERF-binding site ATCTA [29], the bZIP TF binding site, ABA-responsive element (ABRE) core ACGT [30], [31], and the NAC recognition site (NACR), CACG [32], were present in about half of the promoters of filtered genes, whereas another ERF binding site, dehydration-responsive element/C-repeat (DRE/CRT), was only present in one-fifth the promoters of filtered downregulated genes. Moreover, the Homeobox-binding sequence ATTA [33] and the GT-motif bound by MYB TFs, AACCA [9], were present in most of the promoters of filtered genes. Notably, a novel motif, CACGT, that shares the sequence for both ABRE and NACR was present in 50% of the promoters of filtered upregulated genes. Taken together, classification of TFs that are systemic responsive and identification of *cis*-elements from promoters of systemic responsive genes would provide a basis for studying transcriptional regulatory pathways of hypoxic systemic responses.

### Ethylene signaling mediates systemic responses

**Figure 5:**
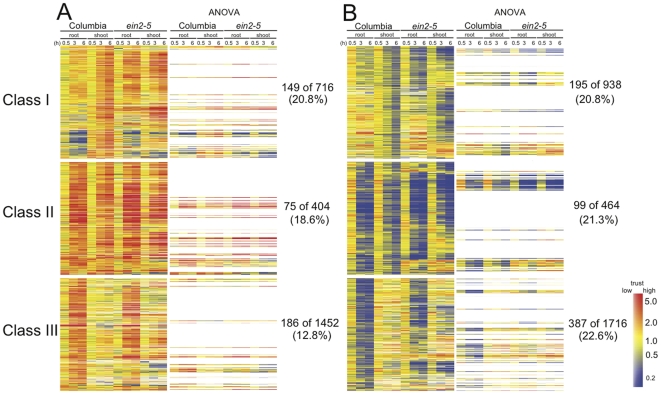
Ethylene-mediated signals controlled certain proportion of hypoxic core responses and hypoxic systemic responses. Fourteen-day-old Columbia and *ein2-5* plants were root flooded for up to 6 hours. Shoot and root tissues were collected at specific time points (0, 0.5, 3, and 6 h). Total RNA was analyzed by microarray analysis with the 0 hour time point as the control channel. Gene Tree Clustering was performed using the GeneSpring software package by Pearson Correlation similarity measure and Average Linkage clustering algorithm from gene lists of each class. Three classes of genes were filtered by >2-fold change (upregulated) or <0.5-fold change (downregulated) expression with a *p* value<0.05 at any one time point from 1 to 12 h in shoot and/or root in Columbia samples. Class I, regulated in shoots only; Class II, regulated in both shoots and roots; Class III, regulated in roots only. A one-way analysis of variance (ANOVA) between Columbia and *ein2-5* was performed using the GeneSpring software package via Welch t-test by *p* value<0.05 cutoff and multiple testing correction with Benjamini and Hochberg False Discovery Rate to identify differentially expressed genes between the two strains. Filtered genes are shown in color in ANOVA columns, while blank indicates genes filtered out. The ratio indicates the number of differentially expressed genes out of the total gene number in each class. The percentage of differentially expressed genes in *ein2-5* in each class is given in parentheses. Color scale indicates treatment-to-control ratios of expression. A lighter color in the scale indicates lower trust with a higher *p* value. Corresponding datasets can be found in Supporting Information File S2.

Gene responses in shoots of root-flooded plants implicates that signals are delivered from roots and cause consequential systemic responses. ACC, the immediate precursor of ethylene, has been shown to function as a signal, which results in petiole epinasty, from roots to shoots in waterlogged tomato plants [5]. To test whether ethylene is involved in mediating systemic transcriptional responses during flooding, we performed root flooding treatment on an *ethylene insensitive 2-5* (*ein2-5*) mutant and observed global changes of gene expression using microarrays. The expression levels of flooding-inducible genes were subjected to a one-way analysis of variance (ANOVA) between Columbia and *ein2-5*. Among Class I genes, 149 of the 716 genes (20.8%) in the upregulated list and 195 of the 938 genes (20.8%) in the downregulated list were significantly different; in the Class II gene list, 75 of the 404 genes (18.6%) in the upregulated list and 99 of 464 genes in the downregulated list were significantly different (Figure 5, File S2). This result supports the fact that a proportion of systemically regulated genes is mediated by ethylene signaling pathways.

To characterize potential functions of genes that are regulated through ethylene-mediated signaling pathways during hypoxia, we identified functional categories with enrichment of differentially expressed genes in *ein2-5* under root flooding (Table 3). In the systemic upregulated gene list, gene induction associated with DNA metabolism, transport, development, and metal handling was significantly affected (*P* value>0.05) in *ein2-5*. In the systemic downregulated gene list, gene repression associated with DNA metabolism, amino acid metabolism, RNA metabolism, and hormone metabolism was significantly affected in (*P* value>0.05) *ein2-5*. These results suggest that ethylene signaling mediates systemic regulation of these functional categories.

**Table 3:** Over-represented functional categories in gene lists that are differentially regulated in *ein2-5*.

		*P* value					
			Upregulated			Downregulated	
		Class I	Class II	Class III	Class I	Class II	Class III
Functional cateogories	Number of genes in category	Shoot only	Both shoot and root	Root only	Shoot only	Both shoot and root	Root only
misc	1595	**3.04E-03**	**4.00E-02**	**4.11E-03**	1.05E-01	**7.19E-07**	**2.86E-03**
DNA	3135	**6.60E-06**	**1.90E-02**	**2.24E-06**	**6.20E-06**	np.	**2.72E-12**
transport	1044	**5.06E-05**	2.09E-01	1.69E-01	1.60E-01	2.28E-01	**1.26E-06**
development	791	**6.16E-03**	np.	**1.90E-02**	1.90E-01	**2.10E-02**	**9.80E-05**
metal handling	89	**6.71E-03**	np.	3.02E-01	3.10E-01	2.03E-01	1.90E-01
amino acid metabolism	258	8.00E-02	**1.80E-02**	2.46E-01	**4.70E-02**	1.36E-01	1.68E-01
gluconeogenesis/glyoxylate cycle	13	np.	**2.80E-02**	np.	np.	np.	np.
signalling	1365	1.31E-01	5.20E-02	**3.60E-02**	1.43E-01	2.00E-01	8.20E-02
cell	824	1.95E-01	np.	**4.60E-02**	5.70E-02	2.62E-01	1.24E-01
RNA	3101	1.11E-01	6.90E-02	**5.38E-03**	**2.20E-02**	5.30E-02	**2.40E-02**
protein	4647	5.30E-02	1.03E-01	**4.20E-02**	8.10E-02	**1.50E-02**	**3.14E-08**
stress	1223	1.15E-01	2.45E-01	**3.80E-02**	1.52E-01	**4.20E-02**	**1.20E-02**
secondary metabolism	442	1.79E-01	1.83E-01	**8.43E-03**	2.19E-01	**8.11E-03**	**2.16E-07**
not assigned	11832	5.10E-02	9.10E-02	6.10E-02	**4.10E-02**	**3.21E-03**	**1.72E-04**
hormone metabolism	534	2.64E-01	8.60E-02	2.29E-01	**4.89E-11**	5.30E-02	**2.64E-07**
cell wall	536	1.25E-01	2.19E-01	9.80E-02	1.36E-01	**1.64E-04**	**5.96E-03**
lipid metabolism	415	1.67E-01	3.70E-01	2.05E-01	2.62E-01	2.22E-01	**1.40E-02**
Biodegradation of Xenobiotics	28	np.	5.90E-02	np.	np.	np.	**3.75E-03**

np. indicates the category is not present in the class of the gene list.

Bold text indicates a significantly over-represented category (with a P value<0.05).

ANOVA filtered genes (Figure 5) from each class were classified with an Arabidopsis web-based classification tool, Functional Classification SuperViewer (Provart and Zhu, 2003) via Mapman category. Functional categories with P values<0.05 in at least one class are shown in the table.

Hormone metabolism and cell wall synthesis were two major categories that harbored a high frequency of genes differentially expressed in *ein2-5* (Table S8). Notably, in the Class I downregulated list, seventeen out of the twenty genes in the functional category of hormone metabolism are involved in auxin response, while the other three genes are related to ethylene response (Table S9), implicating that suppression of auxin responses by ethylene-mediated signaling could play a role in hypoxic systemic signaling. In the functional category of hormone metabolism in Class II and Class III downregulated lists, two out of four genes are involved in JA metabolism in Class II, while seven and eight out of twenty two genes are involved ABA and auxin responses in Class III, respectively, implicating that auxin, ABA and JA might interplay with ethylene signals in hypoxic core responses (Table S9). Moreover, many genes involved in the category of cell wall were primarily downregulated in roots of Columbia, while they were not downregulated in roots of *ein2-5*, suggesting that suppression of cell wall synthesis, degradation and modification could be mediated by ethylene under hypoxia (Table S9). The result is conceptually similar to the quiescence strategy in rice, in which ethylene would mediate *Expansin* expression and cell elongation during submergence [2].

**Figure 6:**
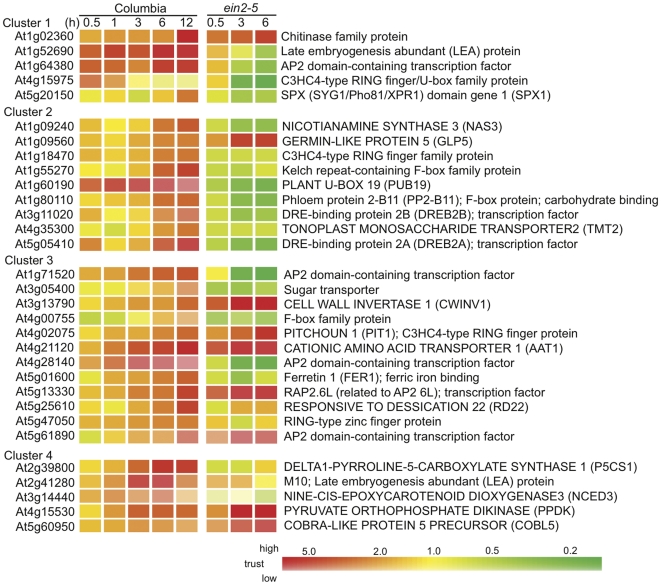
Heatmap of gene expression that are affected in shoot of *ein2-5* during flooding of roots. Genes were selected from the ANOVA filtered lists (Figure 5) in the systemic regulation of carbohydrate management and in functional categories of protein processing, hormone metabolism, RNA metabolism, amino acid metabolism, development, metal handling, and stress. K-means clustering were performed to classified these genes to four clusters based on their systemic expression profiles in the wild-type during flooding using the GeneSpring software package by Similarity Measure Pearson Correlation and one hundred iterations. Color scale indicates treatment-to-control ratios of expression. A lighter color in the scale indicates lower trust with a higher *p* value.

To look into which systemic-responsive genes are mediated by ethylene signal, genes were selected from the ANOVA filtered lists (Figure 5, File S2) in the systemic regulation of carbohydrate management and in several functional categories, including protein processing, hormone metabolism, RNA metabolism, amino acid metabolism, development, metal handling, and stress. These genes were grouped with k-means clustering to four clusters based on their systemic expression profiles in the wild-type during flooding using the GeneSpring software package by Similarity Measure Pearson Correlation and one hundred iterations (Figure 6). In the category of protein processing, genes involved in ubiquitin-dependent protein degradation were the majority. One gene encoding RING finger/U-box protein was in the Cluster 1, two genes encoding RING finger/U-box proteins and two genes encoding F-box proteins were in the Cluster 2, and one gene encoding F-box protein and two encoding RING finger/U-box proteins were in the Cluster 3. This result suggests that induction of ubiquitin-dependent protein degradation could be mediated by ethylene signaling in hypoxic systemic responses. Expectedly, systemic expressions of many hormone related genes were mediated by ethylene. AP2/ERF TFs are known to mediate ethylene, JA, and ABA signaling under stress [34]–[36]. Induction of many AP2/ERF TFs were affected in *ein2-5* in Cluster 1, 2, and 3, including one in Cluster 1, two DRE-binding protein (DREB2A and DREB2B) in Cluster 2, and four in Cluster 3. These indicate that these AP2/ERF TFs could mediate ethylene signals in systemic responses. A *HIGHLY ABA-INDUCED PP2C GENE 1* (*HAI1*) and a gene involved in ABA biosynthesis, *NINE-CIS-EPOXYCAROTENOID DIOXYGENASE 3* (*NCED3*), have a lower induction in *ein2-5*, suggesting involvement of ethylene in ABA signaling and ABA biosynthesis in systemic responses. Regarding genes involved in the systemic regulation of the carbohydrate management, *CELL WALL INVERTASE 1* (*CWINV1*), *PYRUVATE ORTHOPHOSPHATE DIKINASE* (*PPDK*), and two sugar transporters (*TONOPLAST MONOSACCHARIDE TRANSPORTER 2* and At3g05400) were affected in *ein2-5*, suggesting that part of the systemic regulatory program of the carbohydrate management is regulated through ethylene signaling.

**Figure 7:**
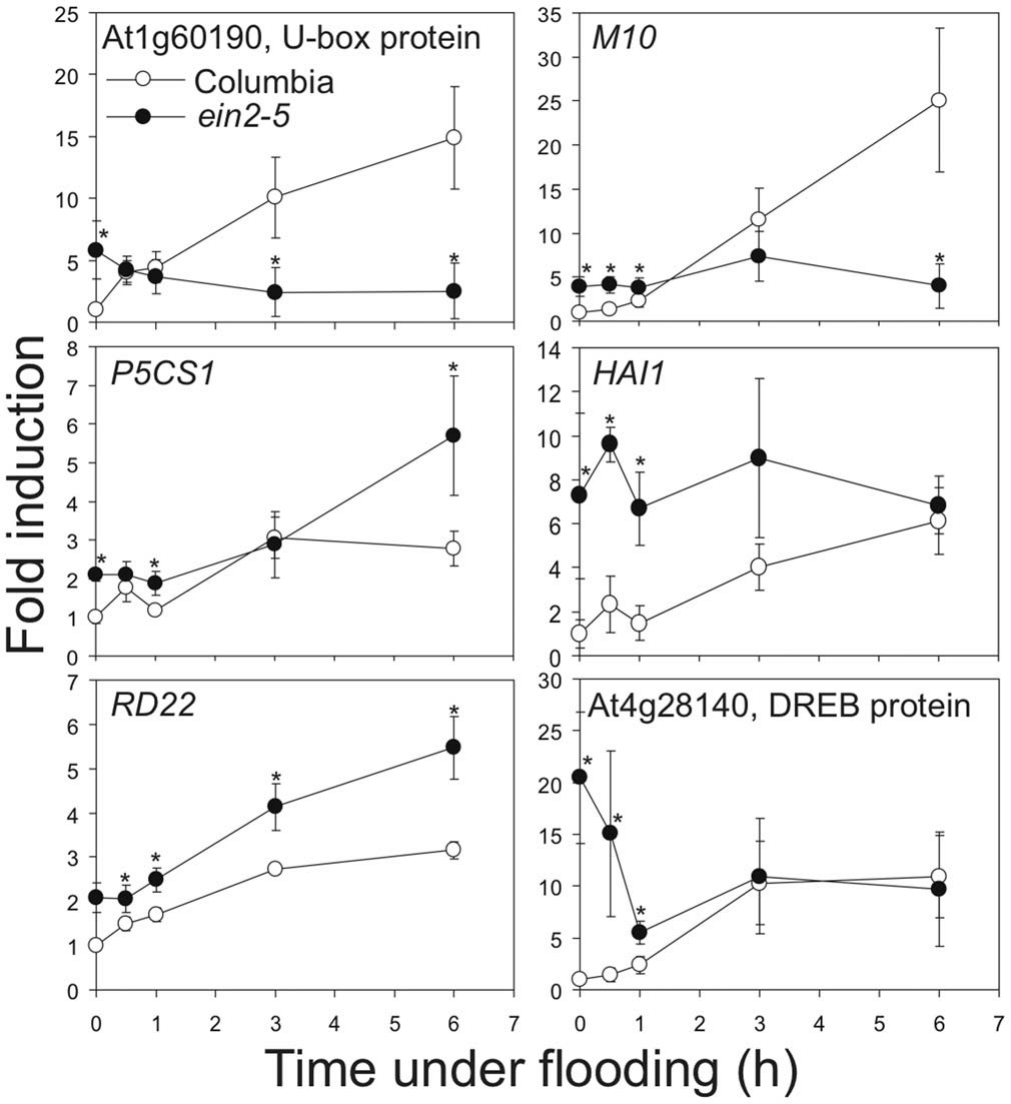
Interactions between hypoxia-derived signal and ethylene-mediated signal in hypoxic systemic responses. Root-flooding-induction of At1g60190 and *M10* expression in shoots was diminished in *ein2-5* mutant. Root-flooding-induction of *DELTA1-PYRROLINE-5-CARBOXYLATE SYNTHASE 1* (*P5CS1*) and *HIGHLY ABA-INDUCED PP2C GENE 1* (*HAI1*) expression in shoots was intensified in *ein2-5* mutant. Basal levels of *RESPONSIVE TO DESSICATION 22* (*RD22*) and At4g28140 were altered in *ein2-5* mutant. Fourteen-day-old Columbia and *ein2-5* plants were root flooded for up to 6 hours. Shoot tissues were collected at specific time points (0, 0.5, 1, 3, and 6 h). Total RNA was analyzed by quantitative RT-PCR using gene-specific primers. The level of *Tubulin* mRNA was used as an internal control. The data represent means ± S.D. from six independent biological replicates, and an asterisk indicates a significant difference between Columbia and *ein2-5* (P<0.05). Gene details are shown in the Supporting Information Table S10.

The differential expression patterns of several systemic-responsive genes in *ein2-5* were determined using Q-RT-PCR. Notably, our analysis showed at least three regulatory programs for systemic responses (Figure 7). Induction of genes such as *M10* [encoding a Late Embryogenesis Abundant (LEA) protein] and At1g60190 (encoding a U-box protein) were ethylene dependent, and suppression of *DELTA1-PYRROLINE-5-CARBOXYLATE SYNTHASE 1* (*P5CS1*) and *HAI1* were ethylene dependent (Figure 7). Other systemic-responsive genes, such as *RESPONSIVE TO DESSICATION 22* (*RD22*) and At4g28140 (encoding a DREB protein), were affected in *ein2-5* with higher basal expression, but still showed similar induction patterns. Interestingly, the induction of *HAI1* in the wild-type plant could be caused by a decrease of ethylene-mediated suppression because the basal level of *HAI1* in *ein2-5* was similar to the highest induction level in the wild-type plant. Regarding the rate of increases, some genes were not induced to the plateau of their induction curves, such as At1g60190, *M10* and *HAI1*, suggesting that longer treatments might be required to induce these genes. Taken together, these data unraveled dynamic roles of ethylene signaling in transcriptional reprogramming of systemic response under root flooding.

Many of these systemic responsive genes that were differential regulated in *ein2-5* mutant are associated with acclimation of other stresses; thus, it is possible that their biochemical functions could help plants to combat stress. Induction levels of several *LEA* genes were decreased in *ein2-5* mutant (Figure 5, 6 and 7, File S2). LEA proteins are abundant in seeds of many plants and also in other organs of plants under stresses, including drought, salt, and cold stresses [37], [38]. Based on amino acid composition, LEA proteins are very hydrophilic, and might protect other proteins from aggregation due to stresses [39]; hence, LEA proteins are speculated as a type of molecular chaperone during stresses or a role in stress tolerance [37]–[40]. Induction of *P5CS1* and *RD22* was affected in *ein2-5* mutant (Figure 5, 6 and 7, File S2). P5CS genes are induced by drought stress and ABA to regulate biosynthesis of proline and to subsequently control proline accumulation for stress acclimation [41]–[44]. *RD22* gene encodes an unknown function protein but it transcriptional regulation is strongly associated with dehydration, salt stress and ABA treatment [45], [46]. These show that systemic induction of genes associated with stress acclimation responses could be affected in *ein2-5* mutant, which suggests that the regulation of ethylene signal might be beneficial for plants under partial hypoxia.

### ABA biosynthesis is regulated differently in shoots and roots to transduce hypoxic signal

**Figure 8:**
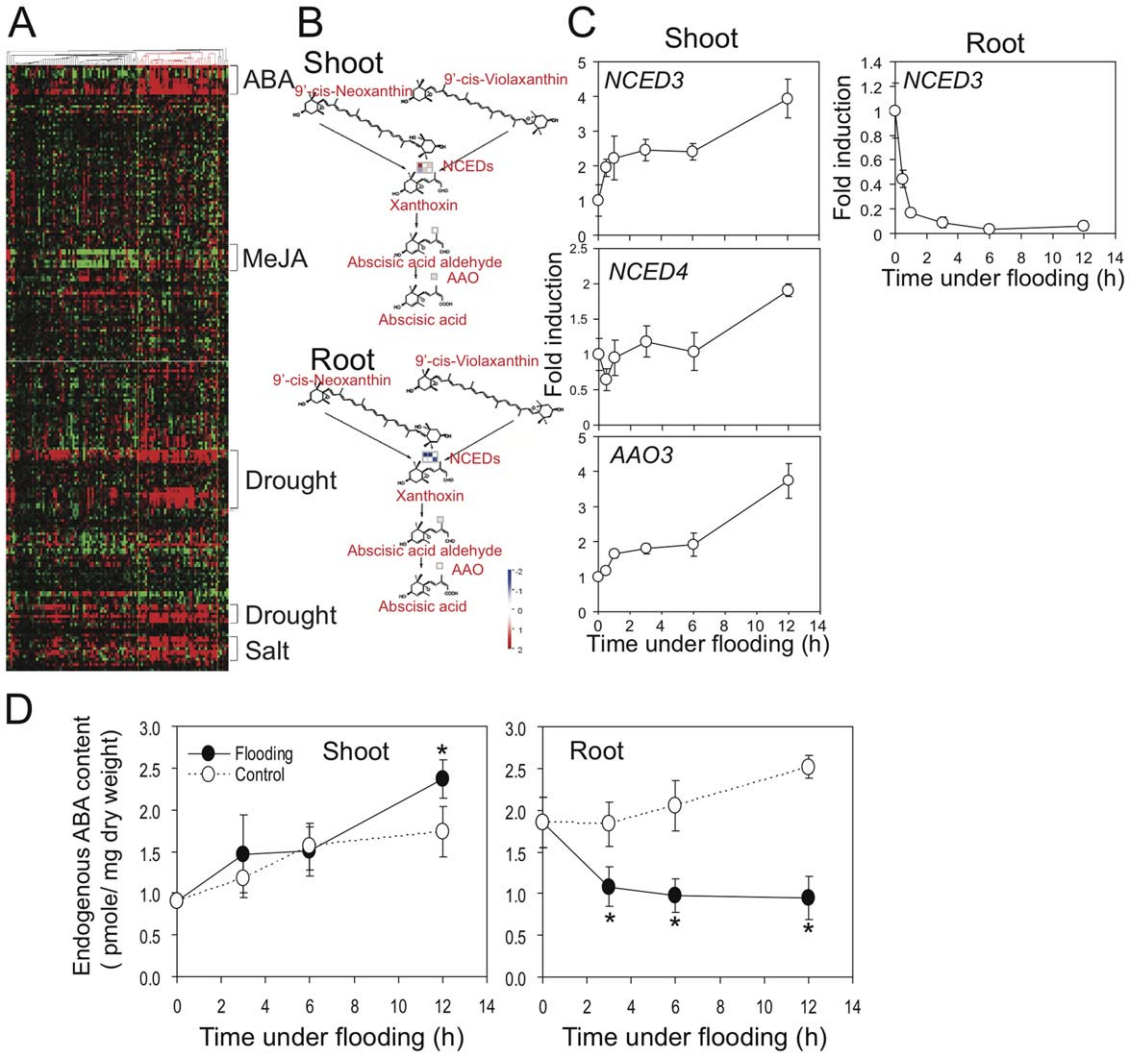
Gene associated with ABA biosynthesis are upregulated in shoots and downregulated in roots under flooding. (A) Many hypoxic systemic responsive genes were ABA-, drought-, and salt-responsive genes. The list of highly induced genes filtered from Class I list (shoot induction only) by showing a >4-fold change in expression with spot intensity >200 at 12 hour flooding time point. A hierarchical clustering was performed via Pearson Correlation distance measurement in Genevestigator. Expression of these hypoxic long-distance induced genes in response to hormones and environmental stimuli were retrieved from Genevestigator database. (B) Genes associated with ABA metabolism were differentially regulated between shoots and roots under flooding of roots, as determined by Mapman software analysis. The Mapman overview of ABA metabolism showed that corresponding genes acted in an opposite manner in shoots and roots. Log_2_ expression values at the 6 h time point for individual genes are plotted as boxes as indicated color in the scale bar. (C) Expression of ABA biosynthesis genes was upregulated in shoot but downregulated in root under root flooding, as determined by Q-RT-PCR. Fourteen-day-old Columbia plants were root flooded for up to 12 hours. Shoot and root tissues were collected at specific time points (0, 0.5, 1, 3, 6, and 12 h). Total RNA was analyzed by microarray (B) and quantitative RT-PCR (C) using gene-specific primers. In Q-RT-PCR, the level of *Tubulin* mRNA was used as an internal control. The data represent means ± S.D. from six independent biological replicates. Gene abbreviations are as follows: *NCED*, *NINE-CIS-EPOXYCAROTENOID DIOXYGENASE*; *AAO3*, *ABSCISIC ALDEHYDE OXIDASE 3*. Gene details are shown in the Supporting Information Table S10. (D) Endogenous ABA contents were slightly increase in shoots but rapidly reduced in roots under roots flooding. Fourteen-day-old Columbia plants were root flooded for up to 12 hours. Seedlings on wet filter paper without flooding were collected at corresponding time points as controls. The data represent means ± S.D. from four independent biological replicates, and an asterisk indicates a significant difference between root flooded and control samples (P<0.05).

Using the Mapman software analysis, we found that the ABA metabolic pathways were distinctly regulated in shoots and roots under root flooding (Figure 8b). *NCED3* that encodes a key enzyme in ABA biosynthesis was highly downregulated in roots under flooding, as assessed by microarray and Q-RT-PCR analyses. By contrast, expression of this gene, its homolog *NCED4*, and that of *AAO3*, which encodes an abscisic aldehyde oxidase, was upregulated in shoots under flooding (Figure 8c). Endogenous ABA levels were further measured in shoots and roots under root flooding. The level of endogenous ABA in roots was dramatically decreased under root flooding, while the level of endogenous ABA in shoots was slightly increased (Figure 8d). These results suggest that ABA biosynthesis is transcriptionally upregulated in shoots and downregulated in roots under flooding.

To determine whether ABA plays any role in the systemic transcriptional induction, we first cross-referenced genes that are systemically induced under flooding to their expression in response to ABA treatment with Genevestigator [47], [48]. A list of genes filtered from the Class I list by showing a >4-fold change in expression with a spot intensity >200 at the time point of 12 h after flooding was analyzed with hierarchical clustering via Pearson Correlation distance measurement in Genevestigator (Figure 8a). Among these genes highly induced in shoots, most were responsive to ABA treatment. Notably, these genes were also responsive to drought and salt stress (Figure 8a). These results suggest that the induction of certain Class I genes during flooding could be mediated by ABA signaling and that this mechanism could be similar to the response to drought and salt stress.

**Figure 9:**
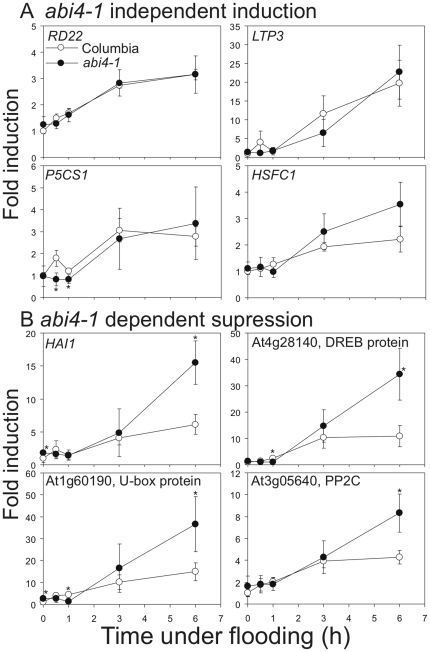
ABA mediation of hypoxic systemic responses. Induction of gene expression under root flooding was unchanged (A) or increased (B) in shoots of the *abi4-1* mutant. Fourteen-day-old Columbia and *abi4-1* plants were root flooded for up to 6 hours. Shoot tissues were collected at specific time points (0, 0.5, 1, 3, and 6 h). Total RNA was analyzed by quantitative RT-PCR using gene-specific primers. The level of *Tubulin* mRNA was used as an internal control. The data represent means ± S.D. from six independent biological replicates, and an asterisk indicates a significant difference between Columbia and *abi4-1* (P<0.05). Gene details are shown in the Supporting Information Table S10. Gene abbreviations are as follows: *RD22*, *RESPONSIVE TO DESSICATION 22*; *LTP3*, *LIPID TRANSFER PROTEIN 3*; *P5CS1*, *DELTA1-PYRROLINE-5-CARBOXYLATE SYNTHASE 1*; *HSFC1*, *HEAT SHOCK FACTOR C1*; *HAI1*, *HIGHLY ABA-INDUCED PP2C GENE 1*.

To further determine how ABA affects the systemic induction of genes under flooding, we analyzed the expression of selected flooding-inducible genes in shoots of an ABA-insensitive mutant, *abi4-1*, by Q-RT-PCR (Figure 9). Because ABA signaling pathways could be affected by ethylene [49], [50], we assessed genes that are affected in shoots of *ein2-5* from the microarray data. Among eight selected genes, four genes were upregulated in *abi4-1*, suggesting an inhibitory role of ABA. The expression of the other four genes was similar to that in Columbia. Although five of these selected genes have been shown to be ABA-responsive genes [42], [43], [51]–[53], in our study, they were either upregulated in *abi4-1*, such as *HAI1* and At3g05640, or expressed similarly in *abi4-1* and wild-type, such as *RD22*, *LIPID TRANSFER PROTEIN 3* (*LTP3*), and *P5CS1*. This result supports the view that hypoxic systemic signals induce expression of certain genes and that ABA could play an inhibitory role to attenuate these inductions.

## Discussion

The long-term goal of this work is to dissect adaptations in non-wetland species under the aspect of systemic responses under root flooding. With the exception of rice, most crops cannot tolerate severe flooding conditions due to their distinct morphological and physiological adaptations. Nevertheless, non-wetland plants should evolve their adaptations to cope with relatively milder flooding conditions, such as root flooding. Under hypoxic conditions, plants readjust large-scale transcriptional and metabolic regulatory programs [15], [16], [18], [54], [55]. When roots are flooded, *Arabidopsis* (Figure 1) and cotton [17] reprogram their transcription globally in both roots and shoots. The transcriptional regulation in shoots indicates the occurrence of systemic responses for an entire plant to cope with partial flooding.

### Local and systemic hypoxic responses in *Arabidopsis* and other plants

By comparing this work with published transcriptomic data in other plant species, we could evaluate conservation and specificity between plant species or between whole-plant hypoxia and root hypoxia conditions. In this work, we defined that local hypoxic responses could be mostly tissue autonomous, which are responsive in the tissues that are directly subjected to hypoxia, either under whole-plant hypoxia or root hypoxia. Regarding local responses across species, a recent article has summarized that regulation of genes associated with anaerobic metabolism, such as glycolysis and fermentation, is highly conserved across higher plants, including *Arabidopsis*, rice, and poplar [14]. The article mainly focuses on the comparison across kingdoms and shows that genes involved in signaling and transcriptional regulation are relative specific within kingdoms [14]. Several higher plant specific hypoxia-responsive unknown proteins are defined in the article as HUPs and classify these HUPs to either *Arabidopsis* specific or conserved in *Arabidopsis*, poplar, and rice [14]. Nevertheless, since functions of these HUPs were not well annotated, this classification would not be able to provide functional information to evaluate conservation and specificity between these species. To identify *Arabidopsis* specific genes, we took advantage of the gene list with genes that are specifically induced in higher plants [14], and we selected those genes that are not induced in rice and poplar (Tables S11, S12). Several functional categories that associated with energy crisis were over-represented in this *Arabidopsis* specific gene list, including major CHO metabolism, lipid metabolism, and amino acid metabolism (Table S12). Several *Arabidopsis* specific genes were related to signaling, such as protein kinase and ubiquitination, and transcriptional regulation (Table S12), suggesting signal regulatory modules across species could be mediated by distinct components. This is also possible that homologous genes with sequence variation for similar functions were evolved across species, so their sequence conservation could not totally reflect their functional conservation; hence, regulation of distinct components could be observed. A gene encoding expansin 10A was induced in *Arabidopsis* but not in poplar or rice (Table S11). Because induction of other expansin members in poplar and rice was observed [18], [56], this could also suggest that distinct sets of homologs were expressed in different plant species. Moreover, a recent publication shows comparison across three dicotyledonous plants and suggests the responses between *Arabidopsis* and cotton are more similar by comparing to poplar, which is a relative flooding tolerant plant [57].

Regarding systemic hypoxic responses, the transcriptomic data in cotton [17] was the only suitable data to compare with *Arabidopsis* data. Rice could not be a suitable model for studying systemic hypoxic responses, because root system of rice is tolerant to hypoxia and most lowland rice is usually grown under root submergence. Poplar may be also not a good material for transcriptomic study. In poplar tree under root waterlogging, transcriptional regulation is mainly observed in roots, but not significant in leaves [18]. To compare systemic responses between *Arabidopsis* and cotton, we took advantage of those genes with significant changes in leaves of waterlogged cotton [17] by comparing expression of their closest homologues in *Arabidopsis* with the expression of our *Arabidopsis* systemic responsive genes. Interestingly, only 157 genes were conserved in both *Arabidopsis* (including 2345 regulated genes) and cotton (including 765 regulated genes) (Figure S2). Among these conserved genes, genes associated with functional categories of protein and stress were over-represented (Table S13), which suggests that translational/post-translational regulation and part of stress related responses in systemic hypoxic responses could be conserved between *Arabidopsis* and cotton. Genes associated in functional categories of cell wall growth and modification, hormone response, and nitrogen metabolism are differentially expressed in leaves of waterlogged cotton [17]. Similarly, these functional categories were over-represented in systemic responsive gene list (in Class I or Class II) in root flooded *Arabidopsis* (Table 1, 2). These functional categories plus categories of amino acid metabolism, nucleotide metabolism, and lipid metabolism were over-represented in both *Arabidopsis* specific and cotton specific list (Table S13). These results suggested that although systemic regulation of many cotton closest homologues in *Arabidopsis* were not conserved between *Arabidopsis* and cotton, regulation of different genes associated with similar functions was conserved. Categories related to carbohydrate metabolisms, such as gluconeogenesis/glyoxylate cycle, major CHO metabolism, oxidative pentose pathway, were relative specific in *Arabidopsis* (Table S13); whereas, only expression of several genes encoding glycosyl hydrolase, glycosyl transferase, and sugar transporter are altered in leaves of cotton [17]. These suggested that the systemic carbohydrate managing program is more comprehensive in *Arabidopsis* than in cotton under similar conditions. We also speculate that the weaker systemic responses of carbohydrate management in cotton could be attributed to its larger plant size. Larger tissues could store more carbohydrates for anaerobic glycolysis and fermentation during hypoxia; hence, the requirement of systemic sources of carbohydrates might be less.

### Existence of a novel systemic communication

We further showed that a systemic carbohydrate managing program is controlled by transcriptional regulation under flooding to form a systemic communication between flooded and non-flooded tissues. As shoots were able to respire under root flooding, shoots were expected to produce additional carbohydrates (Figure 2, Table 1, Table S1, S3, S4). Unlike roots under hypoxia, shoots could primarily induce unidirectional invertases rather than bidirectional sucrose synthases to efficiently degrade sucrose, even though more energy is needed by the invertase route. The small carbohydrates produced in shoots could be from starch catabolism and from fatty acid degradation via glyoxylate cycle and gluconeogenesis (Figure 2). Due to oxygen limitation, roots may require more carbohydrates for anaerobic glycolysis and fermentation to obtain ATP. Notably, the transcriptional regulation for producing carbohydrates occurred in shoots, where the tissues were not directly subjected to hypoxia; nevertheless, the cue to initiate these systemic responses in shoots must be from roots, where the tissues were directly subjected to hypoxia. Ethylene could also play a role in this carbohydrate managing program. Several systemic-responsive genes in this program, including an invertase gene, *PPDK*, and two sugar transporter genes, were affected in *ein2-5* mutant (Figure 6). Taken together, this comprehensive carbohydrate managing program provides an example of how systemic communications could facilitate plants to combat stress.

### Transcriptional regulation and changes of metabolism under flooding

Reprogramming of gene expression under flooding could reveal putative changes of metabolic pathways. Metabolite profiling in several plant species, such as rice, wheat, poplar, and *Arabidopsis*, under low oxygen has been documented [18], [54], [58]. Transcript levels of anaerobic glycolytic and fermentative genes are induced under local hypoxic responses in all of these species, and several conserved and divergent responses on levels of related metabolites across these species can also be found. For example, low level accumulation of glucose-6-P or fructose-6-P is observed in *Arabidopsis* and poplar within 48 hours of low oxygen treatments [18], [54], whereas this can not be detected in rice and wheat after 24 hours of low oxygen treated but in rice after 96 hours of treatment [58]. These suggest that induction of sucrose degradation is conserved across these species, but the rate of glucose-6-P or fructose-6-P accumulation is distinct. Although the accumulation of sucrose degradation metabolites agrees with the induction of *SUS* genes, the accumulation of metabolites requires longer duration (at least 24 hours), while the induction of *SUS* genes only needs around 3 to 6 hours (Figure 3). A common explanation is that the accumulation of metabolite may not only depend on the transcriptional regulation of key genes, but also the rate of production and consumption of the metabolite. This example suggests that reprogramming of gene expression could be an efficient regulation for metabolic pathways; nevertheless, determination of metabolite levels, which would be informative, might not reflect whether the metabolic pathways are regulated. Regarding systemic hypoxic responses, the metabolite profiling in leaves of poplar under waterlogging is the only suitable data so far [18]. Even though the transcriptional reprogramming is not significant in leaves of poplar, changes of metabolites are observed [18]. The level of sucrose does not reduce in roots of waterlogged poplar, but dramatically decreases in leaves; hence, the hypoxic roots have been suggested as a strong sink and the sucrose in leaves could be transported to roots [18]. This phenomenon agrees with our putative carbohydrate managing program that based on gene expression data. Moreover, the emission of acetaldehyde, an oxidation product of ethanol, is increased in leaves of waterlogged poplar, suggesting an existence of the systemic transportation for ethanol from hypoxic roots to aerial leaves [18], [59].

### Identification of systemic responsive TFs under flooding

To investigate transcriptional regulatory pathways, corresponding TFs need to be identified. In addition to TFs that might mediate hypoxic core responses [8], we have identified various sets of systemic responsive TFs (Figure 4). Binding motifs for these systemic responsive TFs are present in the promoters of systemic responsive genes (Tables S6, S7). Coordinated regulation of groups of genes might be mediated by similar sets of TFs. Thus, these results suggest the existence of transcriptional regulatory programs for systemic response under root flooding. Systemic induction of several AP2/ERF transcripts was affected in *ein2-5* mutant (Figure 6 and 7), further suggesting that part of the hypoxic systemic responses could be mediated by ethylene mainly through AP2/ERFs. *MYB2* had been shown to be highly induced to trigger *ADH1* under low oxygen conditions [9]. Using microarrays and Q-RT-PCR, only weak [16] or no [8] induction of *MYB2* by hypoxia has been observed. Herein, our Q-RT-PCR data showed that *MYB2* was not induced in roots but in shoots under flooding (Table S5), suggesting the involvement of *MYB2* in hypoxic systemic response. Classification and identification of these systemic responsive TFs and *cis*-elements may not be able to conclude any transcriptional regulatory pathways at this stage; nevertheless, these results would be informative for further studies for constructing transcriptional regulatory pathways of systemic responses.

### Ethylene is involved in both hypoxic core responses and hypoxic systemic responses

Ethylene is involved in hypoxia adaptations in rice by regulating ERFs, including *Sub1A* in the quiescent strategy [1], [2] and *SK1*/*SK2* in the escape strategy [3]. In *Arabidopsis*, members in a subclass of ERFs affected hypoxic response and tolerance, including a hypoxia-unresponsive ERF, *RAP2.2* [12] and two Hypoxia Responsive ERFs (*HRE*s) [11]. *HRE1* was also induced under ethylene-mediated hypoxia signaling [13]. Yet, although ethylene mediation of hypoxic core response has been shown in rice and *Arabidopsis*, transportation of ACC from roots to shoots in root-flooded tomato was the only report to show that ethylene is involved in mediating hypoxic systemic responses [5]. In this study, we demonstrated that a proportion of core hypoxic responses was regulated by ethylene-mediated signaling pathways (Figure 5) and that a part of hypoxic systemic responses was mediated by ethylene (Figures 5, 6, 7).

It has been suggested that auxin-dependent adventitious and lateral rooting is influenced by ethylene in tomato and petunia, indicating an interplay between ethylene and auxin signaling pathways [60], [61]. Auxin transport that could induce adventitious roots in root flooded tomato was affected by ethylene [62]. Influence of auxin-responsive gene expression by ethylene has also been observed in hypoxic systemic response (Table S9), suggesting a potential role of ethylene and auxin interplay in systemic responses under root flooding.

Ethylene mediated systemic hypoxic responses could be beneficial to plants under root flooding. Although our root flooding conditions were not harsh enough to cause pronounced phenotypes or the death of *Arabidopsis* for indicating plant survival, alteration of gene expression in *ein2* mutants could point out the significance of ethylene in systemic responses to help plant combat root flooding. First, induction of several genes in the predicted carbohydrate managing program were affect in *ein2*, including an invertase gene, *PPDK*, and two sugar transporter genes, suggesting that the systemic carbohydrate managing program requires ethylene. Second, ethylene upregulated genes involved in acclimation to stresses in shoots. For example, systemic induction of *LEA*s, *P5CS1*, and *RD22* were reduced in *ein2-5*. Third, induction of genes involved in signal transduction, such as TFs, U-box proteins, were reduced in *ein2*, suggesting the requirement of ethylene in acclimation to root flooding in shoots.

### ABA biosynthesis and signaling contribute to hypoxic systemic communication

Reduction of ABA levels enhanced internodal elongation in deepwater rice [63] and shoot elongation in *Rumex palustris* [64]. ABA biosynthesis genes, *NCED*s, were downregulated in tissues under submergence in rice [50], *Rumex* [65], and *Arabidopsis* (Figure 8c), suggesting involvement of ABA in core hypoxic response. In hypoxic systemic response, leaf ABA level was increased in root-flooded citrus [7] and bean [66]. Expression of *NCED3*, *NCED4* and *AAO3* increased in shoots of *Arabidopsis* under root flooding (Figure 8c). Interestingly, the systemic induction of *NCED3* was mediated by ethylene (Figure 6). Together, these results indicate that ABA biosynthesis is regulated differently in shoots and roots in hypoxic systemic communication. Additionally, ABA-inducible anoxia tolerance was thought to act in different mechanisms in *Arabidopsis* shoots and roots [67]. The different mechanisms in shoots and roots may provide an alternative explanation of why some ABA-responsive genes were induced in shoots under hypoxia, but were not affected in the *abi4-1* mutant (Figure 9). The different roles that ABA plays in shoots and roots implicate the involvement of different transcriptional regulatory modules. The fact that ABA, drought, and salt treatments were able to upregulate a proportion of genes that were hypoxic systemic responsive (Figure 8a) suggests that part of the hypoxic systemic responses could be controlled by similar modules in ABA, drought and salt responses. In the scope of cellular responses, stomatal closure has long been known as a common response in ABA, drought and salt treatments [68], [69]. Stomatal closure has also been observed in various species when their roots are waterlogged [6], [66], [70], [71]. Therefore, it would be interesting to ask what common components are involved in stomatal closure, ABA, drought, salt, and hypoxic systemic responses.

### Conclusions

This study provides a view from transcriptomic-based data to interpretations for signaling and metabolic regulation that were supported with genetic dissection and bioinformatic cross-reference. We addressed hypoxic systemic response that is a completely novel branch of the field in hypoxic responses. We also showed the involvements of ethylene signaling and ABA biosynthesis in hypoxic systemic responses. We also discussed candidates of components in such mechanisms and provided informative transcriptomic data for public access, but more studies will be needed to dissect the underlying signaling and regulatory components. This also could not be explained solely by transcriptional regulation in concentration of metabolites, such as carbohydrates. Nevertheless, datasets of systemic-responsive genes and transcription factors could be the basis for future studies to construct the signaling pathways. The involvements of ethylene signaling and regulation of ABA biosynthesis in the systemic responses could be the clues to identify molecules that transduce the systemic signals. This relevance in this work could be a basis to develop a new area in the field of hypoxia studies.

## Materials and Methods

### Plant materials, growth conditions, and hypoxic treatments

Experiments were carried out on *Arabidopsis thaliana* ecotype Columbia-0. The ethylene-signaling mutant, *ein2-5*, and the ABA-signaling mutant, *abi4-1*, were obtained from the Arabidopsis Biological Resource Center, Ohio State University, USA. Seeds were surface-sterilized for 20 min in 0.5% of sodium hypochloride and washed at least five times with sterilized water. Seeds were sown on plates with 0.55% of phytagel (Sigma-Aldrich, St. Louis, MO, USA) in half-strength Murashige and Skoog (MS) medium (Duchefa Biochemie BV, Haarlem, Netherlands) containing 0.5% sucrose, and kept at 4°C in the dark for 3 days. Plates were then transferred to a growth chamber, placed vertically, and grown at 22°C under a long photoperiod with 16 h of light and 8 h of darkness for 5 days. The 5-day-old seedlings were transplanted onto fresh plates, and the plates were placed vertically to prevent roots from growing into medium. The transplanted seedlings were grown in the growth chamber until they were 14 days old.

For flooding treatment, in which only roots were submerged, 14-day-old plants were placed between two foamed plastic strips as a floating platform. To minimize the effects of mechanical stress, the platforms with plants were put on filter papers that were wetted with half-strength MS for 1 h in the dark as pre-treatment. The platforms were then transferred to liquid half-strength MS that was bubbled with 3% oxygen balanced with nitrogen for the indicated times. The liquid MS medium was pretreated with 3% oxygen for one hour before use. For non-flooding controls, the platforms stayed on filter paper as it during the pre-treatment, and collected parallel with flooded treatments. For submergence treatment, the plates with 14-day-old plants were submerged into half-strength MS that was bubbled with 3% oxygen balanced with nitrogen for the time indicated.

### RNA extraction and microarray experiments

Total RNA was isolated from shoots or roots using TRIZOL reagent (Invitrogen, Carlsbad, CA, USA) according to the manufacturer's description using a 1∶2 ratio of sample∶TRIZOL reagent. Total RNA was subjected to DNase treatment using the TURBO RNA free kit (Ambion) according to the manufacturer's instructions. Genomic DNA contamination and RNA integrity were tested on an Agilent 2100 Bioanalyzer. The preparation of fluorescently-labeled cDNA and microarray experiments were performed at the DNA Microarray Core Facility, Institute of Plant and Microbial Biology, Academia Sinica, as described on http://ipmb.sinica.edu.tw/microarray/protocol.htm. Arrays used in this study were spotted at the Microarray Core Facility, Institute of Molecular Biology, Academia Sinica, with Qiagen Array-Ready Arabidopsis Oligo Set (V3.0). Four independent biological replicates were performed, of which two included a dye-swap. Array signals were detected and analyzed with Axon GenePix 4000B and GenePix 6.0 (Axon Instruments, Inc., Union City, CA, USA), respectively, then exported as GenePix Result (GPR) files that were imported into GeneSpring 7.3 (Silicon Genetics, Redwood, CA, USA) via LOWESS Normalization. The data discussed in this publication have been deposited in NCBI's Gene Expression Omnibus [72] and are accessible through GEO Series accession number GSE28885 (http://www.ncbi.nlm.nih.gov/geo/query/acc.cgi?acc=GSE28885) for the experimental series and GPL7480 for the array platform.

### Quantitative Reverse Transcription-PCR (Q-RT-PCR)

Two µg of total RNA were reverse transcribed with M-MLV reverse transcriptase (Invitrogen, Carlsbad, CA, USA) and oligo(dT)_16_ primers to generate 100 µl of cDNA according to manufacturer's instructions. Q-RT-PCR was carried out with 1 µl of cDNA, 0.4 µM of each primer, and SYBR Green PCR Master Mix (Applied Biosystems, Foster City, CA, USA) on an ABI 7500 Real-Time PCR machine (Applied Biosystems, Foster City, CA, USA) using default settings. Sequences of primers used can be found in Table S10. For Q-RT-PCR experiments, two technical replicates were performed on each biological replicate. More than six biological repeats were usually used for each data point. *TUB3* (AT5G62700) was used as an internal control for normalization. Relative expression levels were compared by calculating the expression of the gene at a certain time point to a common reference sample from the tissues obtained at time zero.

### Quantification of endogenous ABA content

Liquid nitrogen frozen plant tissues were lyophilized and their dry weights were measured. The dried samples were extracted with solution containing 80% methanol and 2% glacial acetic acid and storage at 4°C in the dark for overnight. The extracts were dried with a vacuum centrifugation concentrator and resuspended with TBS buffer. The ABA concentration in the solution was determined by a Phytodetek ABA Test Kit (Agdia Inc., Elkhart, Indiana, USA) according to manufacturer's instructions.

## Supporting Information

Figure S1 **Mapman overview of transcriptional changes in shoots and roots of root-flooded plants.**
(PDF)

Figure S2 **Venn diagram overlap of systemic hypoxia responsive homologues between** ***Arabidopsis*** **and cotton.**
(PDF)

Table S1 **Upregulated genes in functional categories that are over-represented in shoots under flooding.**
(XLS)

Table S2 **Downregulated genes in functional categories that are over-represented in shoots under flooding.**
(XLS)

Table S3 **Q-RT-PCR validation of differentially regulated genes in functional categories that are over-represented in shoots under flooding.**
(XLS)

Table S4 **Sugar transporters tend to be upregulated in shoots but downregulated in roots under flooding.**
(PDF)

Table S5 **Q-RT-PCR validation of selected transcription factors in each class.**
(XLS)

Table S6 **Over-represented DNA motifs present in promoters of upregulated genes in shoots under flooding.**
(XLS)

Table S7 **Over-represented DNA motifs present in promoters of downregulated genes in shoots under flooding.**
(XLS)

Table S8 **Differentially expressed genes in** ***ein2-5*** **that are over-represented in functional categories of hormone metabolism and cell wall under flooding.**
(XLS)

Table S9 **Mediation of** ***ein2-5*** **in hormone signaling, hormone metabolism, and suppression of cell wall biosynthesis under flooding.**
(PDF)

Table S10 **List of primers used for Q-RT-PCR.**
(XLS)

Table S11 **Upregulated genes specific in** ***Arabidopsis*** **in response to hypoxic local responses.**
(XLS)

Table S12 **Classification of upregulated genes that are specific in** ***Arabidopsis*** **in response to hypoxic local responses.**
(XLS)

Table 13 (XLS)

File S1 **Datasets of gene lists for** **Figure 1** **.**
(XLS)

File S2 **Datasets of gene lists for** **Figure 5** **.**
(XLS)
