# Supplementary material for: Insights into Hypoxic Systemic Responses Based on Analyses of Transcriptional Regulation in Arabidopsis
Source: PLoS One. 2011 Dec 15;6(12):e28888. doi: 10.1371/journal.pone.0028888 (PMC3240646; doi:10.1371/journal.pone.0028888)
Supplement: Figure S1 — Mapman overview of transcriptional changes in shoots and roots of root-flooded plants. (PDF) [file pone.0028888.s001.pdf]

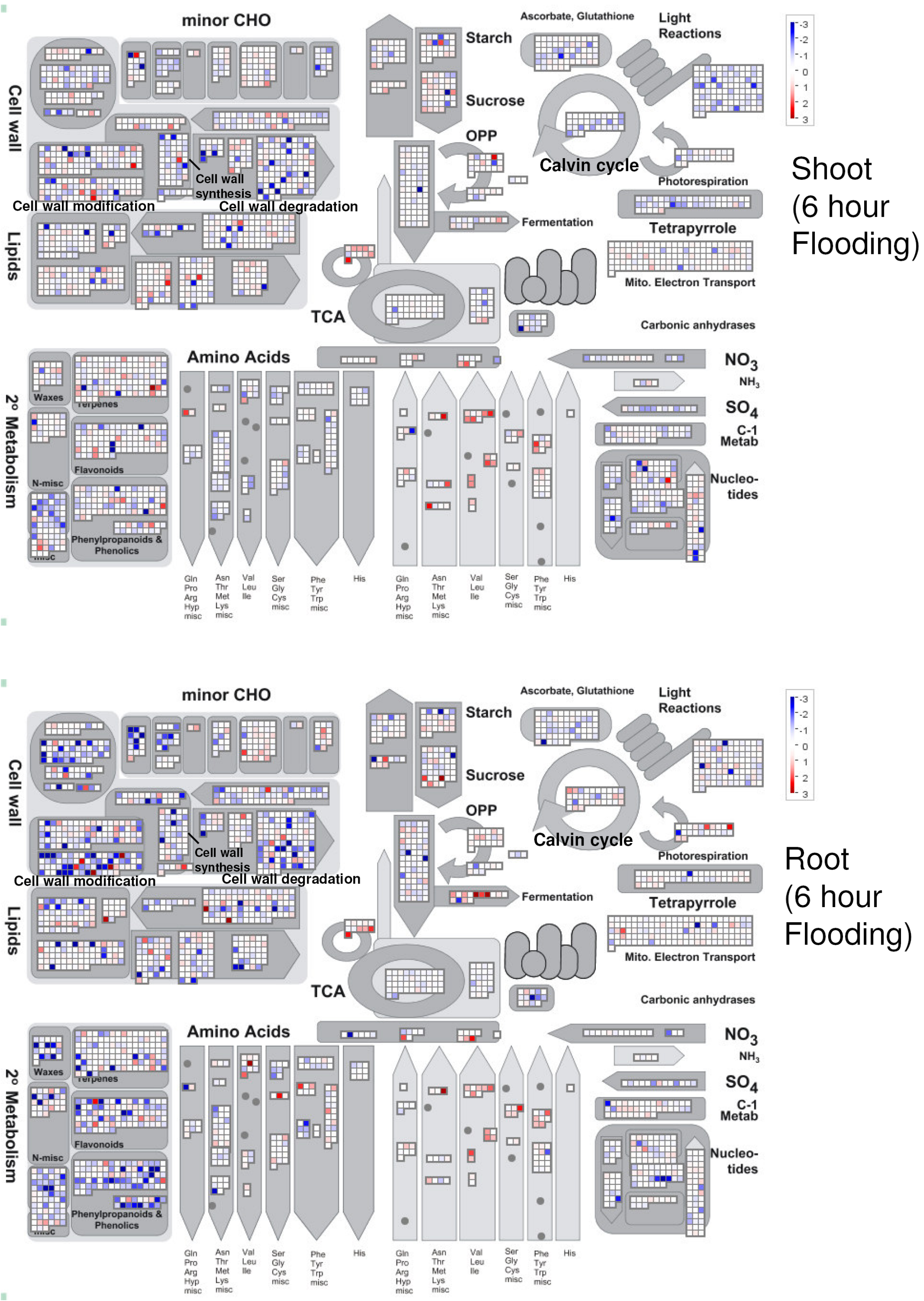


**Figure S1**. Mapman overview of transcriptional changes in shoots and roots of root-flooded plants. The Mapman overview of metabolism showed corresponding genes acted differentially between shoots and roots. Log$_2$ expression values at the 6 hour time point for individual genes were plotted on boxes corresponding to their functional annotation as indicated color in the scale bars.
