# Supplementary material for: Insights into Hypoxic Systemic Responses Based on Analyses of Transcriptional Regulation in Arabidopsis
Source: PLoS One. 2011 Dec 15;6(12):e28888. doi: 10.1371/journal.pone.0028888 (PMC3240646; doi:10.1371/journal.pone.0028888)
Supplement: Figure S2 — Venn diagram overlap of systemic hypoxia responsive homologues between Arabidopsis and cotton. (PDF) [file pone.0028888.s002.pdf]

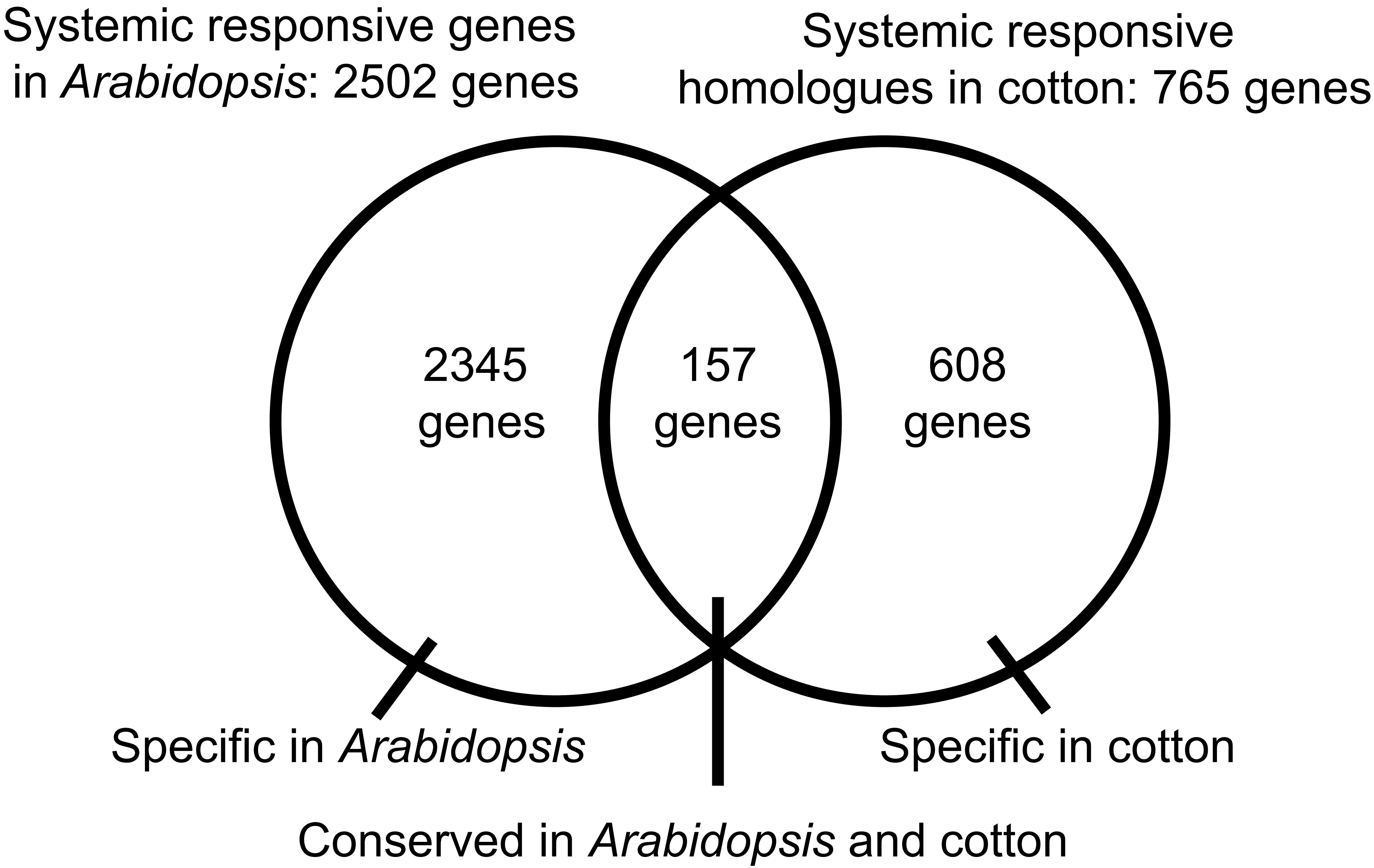


**Figure S2.** Venn diagram overlap of systemic hypoxia responsive homologues between *Arabidopsis* and cotton. Systemic responsive genes were from lists in Class I and II (Figure 1) for *Arabidopsis* and from closest homologues of leaves responsive genes in cotton (Christianson et al., 2010). These lists were filtered out at fold change $> 2$ and $< 0.5$ in expression with p values $< 0.05$ at any one time point from 1 to 12 h in shoots.
