# Supplementary material for: Insights into Hypoxic Systemic Responses Based on Analyses of Transcriptional Regulation in Arabidopsis
Source: PLoS One. 2011 Dec 15;6(12):e28888. doi: 10.1371/journal.pone.0028888 (PMC3240646; doi:10.1371/journal.pone.0028888)
Supplement: Table S4 — Sugar transporters tend to be upregulated in shoots but downregulated in roots under flooding. (PDF) [file pone.0028888.s006.pdf]

Table S4. Sugar transporters tend to upregulated in shoots but downregulated in roots under flooding

Mapman Functional categories	ID	Root 0.5	Root 1	Root 3	Root 6	Root 12	Shoot 0.5	Shoot 1	Shoot 3	Shoot 6	Shoot 12	Description
time under flooding (h)		0.5	1	3	6	12	0.5	1	3	6	12	
**Upregulated :**												
**Shoot only (9 of 716)**												
[34.2] transporter.sugars	At1g20840	1.1	1.4	1.4	1.0	1.4	1.2	1.4	2.0	2.8	3.1	TMT1 (TONOPLAST MONOSACCHARIDE TRANSPORTER1)
[34.2] transporter.sugars	At1g30220	0.6	0.6	0.5	0.5	1.0	1.0	1.3	1.6	2.7	6.0	ATINT2 (INOSITOL TRANSPORTER 2)
[34.2] transporter.sugars	At2g22730	1.0	1.1	1.5	1.7	1.7	0.9	1.1	1.0	1.4	2.1	transporter-related
[34.2] transporter.sugars	At3g05400	0.9	0.9	1.0	1.0	1.0	1.0	1.0	1.1	1.5	2.6	sugar transporter, putative
[34.2] transporter.sugars	At4g02050	0.9	1.3	1.3	1.2	1.1	1.0	1.1	1.6	1.7	2.2	sugar transporter, putative
[34.2] transporter.sugars	At4g35300	0.8	0.6	0.7	0.8	0.9	1.0	0.9	1.1	1.7	2.2	TMT2 (TONOPLAST MONOSACCHARIDE TRANSPORTER2)
[34.2] transporter.sugars	At5g26340	1.0	1.1	1.0	0.5	0.5	1.4	1.7	2.7	4.0	5.1	MSS1 (SUGAR TRANSPORT PROTEIN 13)
[34.2] transporter.sugars	At5g27350	0.9	1.4	1.3	1.3	1.4	1.1	1.1	1.6	2.2	3.1	SFP1; carbohydrate transporter/ sugar porter
[34.8] transport.metabolite transporters at the envelope membrane	At1g61800	1.3	1.9	2.3	2.1	1.4	0.9	0.8	1.3	2.7	5.0	GPT2 (glucose-6-phosphate/phosphate translocator 2)
**Shoot & root both (1 of 404)**												
[34.99] transport.misc	At1g79360	2.3	3.8	2.9	1.4	1.7	0.8	1.7	3.1	7.0	4.6	transporter-related
**Root only (4 of 1452)**												
[34.2] transporter.sugars	At1g54730	1.0	1.2	2.5	2.6	2.7	0.9	1.2	1.4	1.4	1.5	sugar transporter, putative
[34.2] transporter.sugars	At2g20780	1.1	1.0	1.4	1.8	2.6	1.1	0.8	0.8	1.0	1.1	mannitol transporter, putative
[34.2] transporter.sugars	At3g19930	1.3	2.8	3.3	2.3	2.0	1.0	1.1	1.1	1.1	1.3	STP4 (SUGAR TRANSPORTER 4)
[34.11] transport.NDP-sugars at the ER	At2g02810	1.1	1.6	2.6	2.1	1.4	1.1	1.1	1.5	1.2	1.9	ATUTR1/UTR1 (UDP-GALACTOSE TRANSPORTER 1)
**Down-regulated:**												
**Shoot only (1 of 938)**												
[34.2] transporter.sugars	At5g59250	1.00	1.03	0.94	0.64	0.51	1.08	1.00	0.97	0.72	0.37	sugar transporter family protein
**Shoot &root both (4 of 464)**												
[34.2] transporter.sugars	At1g19450	1.14	0.70	0.28	0.21	0.16	1.10	0.99	0.54	0.32	0.28	integral membrane protein, putative
[34.7] transport.phosphate	At2g29650	0.76	0.86	0.50	0.36	0.35	0.91	0.97	0.81	0.57	0.23	inorganic phosphate transporter, putative
[34.8] transport.metabolite transporters at the envelope membrane	At5g17630	0.95	0.79	0.48	0.45	0.44	0.92	0.85	0.66	0.54	0.46	glucose-6-phosphate/phosphate translocator, putative
[34.99] transport.misc	At1g16370	1.42	1.20	0.33	0.18	0.15	1.43	0.84	0.33	0.20	0.19	transporter-related
**Root only (12 of 1716)**												
[34.2] transporter.sugars	At1g08890	0.96	0.94	0.41	0.24	0.17	1.00	1.18	1.26	1.18	1.19	sugar transporter family protein
[34.2] transporter.sugars	At1g08920	1.13	0.62	0.42	0.40	0.49	1.06	1.05	1.12	1.50	1.85	carbohydrate transporter/ sugar porter
[34.2] transporter.sugars	At1g73220	0.94	0.75	0.34	0.13	0.25	1.18	1.33	2.22	2.18	2.53	sugar transporter family protein
[34.2] transporter.sugars	At1g79410	0.69	0.36	0.37	0.53	0.63	1.13	1.16	1.21	1.72	1.50	transporter-related
[34.2] transporter.sugars	At2g18480	0.75	0.51	0.55	0.64	0.28	1.14	0.80	0.72	0.91	0.51	mannitol transporter, putative
[34.2] transporter.sugars	At3g03090	1.22	0.93	0.71	0.53	0.45	0.94	0.85	0.92	1.13	1.05	sugar transporter family protein
[34.2] transporter.sugars	At3g20460	0.55	0.48	0.34	0.28	0.23	1.37	0.92	1.10	0.43	0.71	sugar transporter, putative
[34.2] transporter.sugars	At5g18840	0.88	1.27	1.49	0.74	0.43	1.02	1.57	1.67	0.97	0.88	sugar transporter, putative
[34.2.1] transporter.sugars.sucrose	At1g66570	1.05	1.03	0.50	0.39	0.37	1.18	0.93	0.74	0.63	0.59	ATSUC7 (SUCROSE-PROTON SYMPORTER 7)
[34.7] transport.phosphate	At2g38060	1.30	1.21	0.36	0.18	0.22	1.45	1.34	1.11	0.59	1.21	transporter-related
[34.8] transport.metabolite transporters at the envelope membrane	At1g43310	1.14	1.00	0.63	0.49	0.52	1.12	0.88	0.82	0.89	0.83	triose phosphate/phosphate translocator-related
[34.8] transport.metabolite transporters at the envelope membrane	At5g33320	0.97	0.92	0.56	0.44	0.50	0.89	0.77	0.74	0.74	0.72	CUE1 (CAB UNDEREXPRESSED 1); triose-phosphate transporter
[34.98] transporter.membrane system	At3g47420	0.28	0.26	0.38	0.55	0.54	0.75	0.89	0.83	0.61	0.60	glycerol-3-phosphate transporter, putative

Flooding/ control

Color indicates expression by following scale: 0.25 | 0.5 | 1 | 2 | 4
