# Supplementary material for: Insights into Hypoxic Systemic Responses Based on Analyses of Transcriptional Regulation in Arabidopsis
Source: PLoS One. 2011 Dec 15;6(12):e28888. doi: 10.1371/journal.pone.0028888 (PMC3240646; doi:10.1371/journal.pone.0028888)
Supplement: Table S9 — Mediation of ein2-5 in hormone signaling, hormone metabolism, and suppression of cell wall biosynthesis under flooding. (PDF) [file pone.0028888.s011.pdf]

Table S9. Mediation of *ein2-5* in hormone-signaling and -metabolism and in suppression of cell wall biosynthesis under flooding.

		Flooding/ control												
		Columbia						*ein2-5*						
		Root			Shoot			Root			Shoot			
Mapman bins/ Classification	ID (h)	0.5	3	6	0.5	3	6	0.5	3	6	0.5	3	6	Description
**Hormone metabolism**														
**Class I (20 of 195)**														
[17.2.3] hormone metabolism.auxin.induced-regulated-responsive-activated	At1g29430	0.67	1.22	1.33	0.97	0.52	0.29	0.28	0.25	0.82	1.06	1.66	1.85	auxin-responsive family protein
[17.2.3] hormone metabolism.auxin.induced-regulated-responsive-activated	At1g29440	1.19	2.66	1.37	0.90	0.51	0.32	0.73	1.69	1.78	1.21	1.72	1.71	auxin-responsive family protein
[17.2.3] hormone metabolism.auxin.induced-regulated-responsive-activated	At1g29450	0.98	1.04	1.16	1.00	0.74	0.52	0.22	1.32	1.84	1.19	2.27	2.57	auxin-responsive protein, putative
[17.2.3] hormone metabolism.auxin.induced-regulated-responsive-activated	At1g29490	0.74	1.28	1.24	0.86	0.63	0.45	0.11	0.54	0.92	1.18	2.23	2.72	auxin-responsive family protein
[17.2.3] hormone metabolism.auxin.induced-regulated-responsive-activated	At1g29500	0.96	1.25	1.44	0.96	0.59	0.39	0.43	0.59	0.84	1.32	2.73	2.56	auxin-responsive protein, putative
[17.2.3] hormone metabolism.auxin.induced-regulated-responsive-activated	At1g29510	0.60	1.20	1.31	0.85	0.53	0.36	0.33	0.44	0.41	1.25	1.84	1.72	auxin-responsive protein, putative
[17.2.3] hormone metabolism.auxin.induced-regulated-responsive-activated	At1g75580	0.74	2.13	1.80	0.72	0.84	0.81	0.52	1.13	1.44	1.25	0.97	1.22	auxin-responsive protein, putative
[17.2.3] hormone metabolism.auxin.induced-regulated-responsive-activated	At4g36110	1.41	2.15	2.38	0.82	0.50	0.55	0.74	1.68	2.35	1.61	1.70	1.67	auxin-responsive protein, putative
[17.2.3] hormone metabolism.auxin.induced-regulated-responsive-activated	At4g38840	0.94	1.41	0.67	0.84	0.57	0.51	0.11	1.25	2.15	0.87	1.56	1.58	auxin-responsive protein, putative
[17.2.3] hormone metabolism.auxin.induced-regulated-responsive-activated	At4g38850	1.23	0.25	0.05	1.05	0.68	0.53	0.47	1.18	0.55	1.00	2.38	1.56	SAUR_AC1 (SMALL AUXIN UP RNA 1)
[17.2.3] hormone metabolism.auxin.induced-regulated-responsive-activated	At5g18010	0.98	0.85	0.81	1.09	0.73	0.68	1.08	0.83	0.77	1.18	2.52	2.71	auxin-responsive protein, putative
[17.2.3] hormone metabolism.auxin.induced-regulated-responsive-activated	At5g18020	1.20	1.02	1.40	1.06	0.76	0.59	1.09	0.87	0.98	1.30	3.24	3.55	auxin-responsive protein, putative
[17.2.3] hormone metabolism.auxin.induced-regulated-responsive-activated	At5g18030	1.52	1.25	1.04	1.11	0.76	0.64	1.49	1.38	0.93	1.45	3.19	3.06	auxin-responsive protein, putative
[17.2.3] hormone metabolism.auxin.induced-regulated-responsive-activated	At5g18060	0.97	1.09	0.99	1.02	0.79	0.72	0.24	0.77	0.82	1.27	3.64	3.82	auxin-responsive protein, putative
[17.2.3] hormone metabolism.auxin.induced-regulated-responsive-activated	At5g18080	0.80	0.74	0.75	0.99	0.71	0.57	0.76	0.31	0.85	1.24	2.91	3.62	auxin-responsive protein, putative
[17.2.3] hormone metabolism.auxin.induced-regulated-responsive-activated	At5g27780	0.89	1.56	1.28	0.86	0.51	0.30	0.81	0.78	1.33	1.21	1.78	1.60	auxin-responsive family protein
[17.2.2] hormone metabolism.auxin.signal transduction	At1g70940	0.84	0.85	0.85	0.53	0.79	0.48	1.08	0.76	0.72	1.10	2.63	2.89	PIN3 (PIN-FORMED 3); auxin:hydrogen symporter/ transporter
[17.5.2] hormone metabolism.ethylene.signal transduction	At1g06160	0.73	2.46	2.68	0.49	0.42	0.33	0.74	2.10	2.97	0.96	1.82	1.69	ethylene-responsive factor, putative
[17.5.2] hormone metabolism.ethylene.signal transduction	At2g40940	0.90	1.12	0.79	0.72	0.68	0.43	0.95	1.78	1.60	1.09	1.47	2.00	ERS1 (ETHYLENE RESPONSE SENSOR 1); receptor
[17.5.2] hormone metabolism.ethylene.signal transduction	At5g61590	1.62	4.55	4.24	0.69	0.62	0.42	1.02	5.13	4.57	0.91	1.20	1.38	AP2 domain-containing transcription factor family protein
**Class II (4 of 99)**														
[17.2.2] hormone metabolism.auxin.signal transduction	At1g23080	0.82	0.50	0.40	0.71	0.93	0.71	1.04	0.84	0.65	0.67	1.19	1.12	PIN7 (PIN-FORMED 7)
[17.3.1.1.1] hormone metabolism.brassinosteroid.synthesis-degradation.BRs.DET2	At5g16010	0.82	0.22	0.05	0.93	0.67	0.39	2.07	1.41	0.36	2.10	1.69	0.69	3-oxo-5-alpha-steroid 4-dehydrogenase family protein
[17.7.1.5] hormone metabolism.jasmonate.synthesis-degradation.12-Oxo-PDA-reductase	At2g06050	1.77	0.54	0.52	1.25	0.32	0.24	0.54	0.12	0.11	1.04	0.13	0.05	OPR3 (OPDA-REDUCTASE 3)
[17.7.1.2] hormone metabolism.jasmonate.synthesis-	At1g17420	2.75	0.29	0.11	1.52	0.18	0.18	0.77	0.08	0.04	0.94	0.12	0.03	LOX3 (Lipoxygenase 3); iron ion binding / lipoxygenase
**Class III (22 of 387)**														
[17.1.3] hormone metabolism.abscisic acid.induced-regulated-responsive-activated	At2g47770	0.67	0.12	0.08	1.88	5.92	17.84	2.39	1.60	0.69	1.60	8.24	9.86	benzodiazepine receptor-related
[17.1.3] hormone metabolism.abscisic acid.induced-regulated-responsive-activated	At3g02480	1.59	0.81	0.38	3.98	2.87	8.65	1.74	1.62	1.43	1.51	1.31	2.24	ABA-responsive protein-related
[17.1.3] hormone metabolism.abscisic acid.induced-regulated-responsive-activated	At5g59220	0.58	0.18	0.11	1.97	4.13	9.83	2.41	0.69	0.58	1.77	0.78	0.62	protein phosphatase 2C, putative / PP2C, putative
[17.1.1.1.10] hormone metabolism.abscisic acid.synthesis-degradation.synthesis.9-cis-epoxycarotenoid dioxygenase	At1g78390	0.55	0.32	0.34	1.01	1.88	1.10	1.09	0.74	0.57	1.09	2.61	1.81	NCED9 (NINE-CIS-EPOXYCAROTENOID DIOXYGENASE 9)
[17.1.2] hormone metabolism.abscisic acid.signal transduction	At4g26080	0.41	0.40	0.42	0.68	1.32	1.62	1.48	0.84	0.86	1.37	1.27	1.32	ABI1 (ABA INSENSITIVE 1); calcium ion binding / protein phosphatase type 2C
[17.1.2] hormone metabolism.abscisic acid.signal transduction	At4g34000	0.52	0.34	0.33	0.95	2.04	3.06	0.89	0.73	0.71	0.76	1.19	1.40	ABF3/DPBF5 (ABSCISIC ACID RESPONSIVE ELEMENTS-BINDING FACTOR 3)
[17.1.2] hormone metabolism.abscisic acid.signal transduction	At5g57050	0.50	0.17	0.22	0.91	1.90	3.61	1.36	0.59	0.59	1.52	1.45	1.33	ABI2 (ABA INSENSITIVE 2); protein phosphatase type 2C
[17.2.1.1001] hormone metabolism.auxin.synthesis-degradation	At1g24100	0.97	0.57	0.59	0.91	0.57	0.55	0.50	0.26	0.35	0.69	0.67	0.44	UGT74B1 (UDP-glucosyl transferase 74B1)
[17.2.3] hormone metabolism.auxin.induced-regulated-responsive-activated	At1g28130	0.39	0.17	0.21	1.03	0.79	0.61	0.63	0.58	0.69	1.19	1.19	1.29	GH3.17; indole-3-acetic acid amido synthetase
[17.2.3] hormone metabolism.auxin.induced-regulated-responsive-activated	At3g61750	0.74	0.27	0.23	0.80	0.56	0.53	1.01	0.49	0.70	0.65	0.83	0.52	auxin-responsive protein -related
[17.2.3] hormone metabolism.auxin.induced-regulated-responsive-activated	At5g10990	0.30	0.28	0.33	0.81	1.58	1.70	0.75	0.42	0.49	0.95	1.04	0.77	auxin-responsive family protein
[17.2.3] hormone metabolism.auxin.induced-regulated-responsive-activated	At5g47530	0.52	0.11	0.10	1.10	0.36	1.17	1.05	0.33	0.51	0.07	1.37	0.83	auxin-responsive protein, putative
[17.2.3] hormone metabolism.auxin.induced-regulated-responsive-activated	At5g53590	0.77	0.59	0.46	1.56	1.03	0.86	1.48	1.25	1.11	1.17	0.77	0.58	auxin-responsive family protein
[17.2.3] hormone metabolism.auxin.induced-regulated-responsive-activated	At5g54510	0.75	0.56	0.46	0.89	1.69	1.49	1.24	1.10	0.77	1.15	2.64	3.48	DFL1 (DWARF IN LIGHT 1); indole-3-acetic acid amido synthetase
[17.2.2] hormone metabolism.auxin.signal transduction	At1g23080	0.75	0.46	0.41	0.66	0.96	0.73	1.09	0.87	0.78	0.62	1.17	1.04	PIN7 (PIN-FORMED 7); auxin:hydrogen symporter/ transporter
[17.3.1.2.99] hormone metabolism.brassinosteroid.synthesis-degradation.sterols.other	At5g24140	1.04	0.45	0.32	1.70	1.47	1.19	2.33	2.38	1.22	2.68	3.82	2.49	SQP2 (Squalene monooxygenase 2); oxidoreductase
[17.4.1.1003] hormone metabolism.cytokinin.synthesis-degradation	At2g41510	0.62	0.47	0.19	0.95	0.83	0.57	1.21	1.90	0.64	0.81	1.56	0.79	ATCKX1/CKX1 (CYTOKININ OXIDASE/DEHYDROGENASE 1)
[17.4.1.1003] hormone metabolism.cytokinin.synthesis-degradation	At5g05870	0.73	0.47	0.47	0.86	0.97	0.97	1.12	1.07	1.23	1.20	1.04	1.08	UGT76C1 (UDP-GLUCOSYL TRANSFERASE 76C1)
[17.5.1.1002] hormone metabolism.ethylene.synthesis-degradation	At5g12270	1.23	0.61	0.39	1.10	0.93	1.29	1.51	3.23	1.11		0.49	1.26	oxidoreductase, 2OG-Fe(II) oxygenase family protein
[17.5.1.1002] hormone metabolism.ethylene.synthesis-degradation	At5g59530	0.83	0.66	0.49	0.99	0.94	0.66	1.52	1.77	1.42	1.25	0.82	0.51	2-oxoglutarate-dependent dioxygenase, putative
[17.6.1.12] hormone metabolism.gibberelin.synthesis-degradation.GA3	At1g80340	0.44	0.28	0.29	0.93	0.44	1.41	0.66	0.68	0.46	0.95	0.34	1.12	GA4H (gibberellin 3 beta-hydroxylase); gibberellin 3-beta-dioxygenase
[17.7.3] hormone metabolism.jasmonate.induced-regulated-responsive-activated	At5g48180	1.11	0.80	0.42	1.03	2.11	2.73	2.19	2.24	1.30	1.35	2.29	2.45	kelch repeat-containing protein
**Cell wall**														
**Class I (1 of 195)**														
[10.6.3] cell wall.degradation.pectate lyases and polygalacturonases	At5g63180	0.50	0.94	0.97	0.70	0.64	0.45	1.32	0.75	0.37	0.91	1.24	1.12	pectate lyase family protein
**Class II (8 of 99)**														
[10.2.1] cell wall.cellulose synthesis.cellulose synthase	At1g02730	0.84	0.38	0.70	1.25	0.62	0.45	0.59	0.22	0.27	0.50	0.23	0.16	ATCSLD5 (Cellulose synthase-like D5)
[10.2.1] cell wall.cellulose synthesis.cellulose synthase	At4g39350	1.10	0.37	0.57	0.90	0.55	0.46	0.67	0.19	0.21	0.72	0.49	0.33	CESA2 (CELLULASE SYNTHASE 2)
[10.2] cell wall.cellulose synthesis	At5g03760	0.77	0.24	0.35	0.85	0.57	0.56	0.68	0.14	0.16	0.54	0.30	0.16	ATCSLA09 (RESISTANT TO AGROBACTERIUM TRANSFORMATION 4)
[10.5.1] cell wall.cell wall proteins.AGPs	At3g13520	0.96	0.44	0.21	1.03	0.55	0.57	1.94	0.84	0.59	1.33	1.22	0.79	AGP12 (ARABINOGALACTAN PROTEIN 12)
[10.5.1] cell wall.cell wall proteins.AGPs	At5g44130	1.78	2.67	0.69	0.92	0.64	0.38	1.58	6.19	4.37	1.10	2.07	1.36	fasciclin-like arabinogalactan-protein, putative
[10.6.3] cell wall.degradation.pectate lyases and polygalacturonases	At1g70370	1.49	0.23	0.31	1.55	0.61	0.41	0.79	0.15	0.11	0.71	0.21	0.17	BURP domain-containing protein / polygalacturonase, putative
[10.6.2] cell wall.degradation.mannan-xylose-arabinose-fucose	At1g02640	1.21	0.77	0.55	1.07	1.04	0.66	0.92	1.91	1.03	1.03	1.55	1.50	BXL2 (BETA-XYLOSIDASE 2); hydrolase
[10.8.1] cell wall.pectin*esterases.PME	At1g41830	1.09	0.44	0.51	1.01	0.45	0.45	0.87	0.33	0.22	0.69	0.28	0.18	SKS6 (SKU5 Similar 6); pectinesterase
**Class III (13 of 387)**														
[10.1.9] cell wall.precursor synthesis.MUR4	At4g20460	0.76	0.94	0.62	1.00	1.08	0.85	1.66	3.78	2.47	4.94	8.31	5.51	NAD-dependent epimerase/dehydratase family protein
[10.1.2] cell wall.precursor synthesis.UGE	At4g23920	0.84	0.36	0.28	1.42	1.06	1.27	1.54	0.74	0.58	0.77	0.77	0.50	UGE2 (UDP-D-glucose/UDP-D-galactose 4-epimerase 2)
[10.3.2] cell wall.hemicellulose synthesis.glucuronoxylan	At1g19300	1.04	0.63	0.36	1.50	0.74	0.90	0.66	0.25	0.18	0.86	0.31	0.16	GATL1/GLZ1/PARVUS (Galacturonosyltransferase-like 1)
[10.5.1] cell wall.cell wall proteins.AGPs	At2g20520	0.63	0.28	0.18	1.12	1.03	1.13	2.23	0.78	0.75	1.03	0.66	0.65	FLA6 (FLA6)
[10.6.3] cell wall.degradation.pectate lyases and polygalacturonases	At4g22090	0.67	0.32	0.26	1.55	0.32	0.93	1.70	1.29	0.52		2.33	1.10	pectate lyase family protein
[10.6.3] cell wall.degradation.pectate lyases and polygalacturonases	At4g23500	0.86	0.68	0.51	1.05	1.03	1.01	1.11	1.04	1.05	1.05	1.01	1.12	glycoside hydrolase family 28 protein / polygalacturonase (pectinase)
[10.6.3] cell wall.degradation.pectate lyases and polygalacturonases	At5g06860	1.13	0.67	0.32	1.29	1.51	1.24	1.24	1.98	1.09	1.11	1.78	1.97	PGIP1 (POLYGALACTURONASE INHIBITING PROTEIN 1)
[10.7] cell wall.modification	At1g14720	0.58	0.46	0.33	0.96	0.92	0.69	0.94	1.28	1.04	1.22	2.00	1.60	XTR2 (XYLOGLUCAN ENDOTRANSGLYCOSYLASE RELATED 2)
[10.7] cell wall.modification	At1g62980	0.57	0.38	0.31	1.82	1.40	1.90	1.10	0.67	0.47	1.32	0.80	0.56	ATEXPA18 (ARABIDOPSIS THALIANA EXPANSIN A18)
[10.7] cell wall.modification	At1g69530	0.46	0.73	0.43	0.64	1.20	1.04	0.94	1.27	1.55	0.69	1.24	1.71	ATEXPA1 (ARABIDOPSIS THALIANA EXPANSIN A1)
[10.7] cell wall.modification	At2g37640	0.84	0.57	0.34	0.83	1.16	1.27	2.43	1.78	1.13	1.12	1.22	1.41	ATEXPA3 (ARABIDOPSIS THALIANA EXPANSIN A3)
[10.7] cell wall.modification	At2g40610	0.35	0.60	0.45	0.63	1.76	1.96	0.64	1.08	1.70	0.43	1.05	4.38	ATEXPA8 (ARABIDOPSIS THALIANA EXPANSIN A8)
[10.8.1] cell wall.pectin*esterases.PME	At3g10710	0.49	0.38	0.30	2.20	1.00	2.48	0.92	0.67	0.52	3.00	1.79	2.12	pectinesterase family protein

Color indicates flooding/control expression by following heatmap scale: 0.25 | 0.5 | 1 | 2 | 4

Genes are extracted from downregulation lists.
